# Gridded population mapping for Germany based on building density, height and type from Earth Observation data using census disaggregation and bottom-up estimates

**DOI:** 10.1371/journal.pone.0249044

**Published:** 2021-03-26

**Authors:** Franz Schug, David Frantz, Sebastian van der Linden, Patrick Hostert

**Affiliations:** 1 Geography Department, Humboldt-Universität zu Berlin, Berlin, Germany; 2 Integrated Research Institute on Transformations of Human-Environment Systems, Humboldt-Universität zu Berlin, Berlin, Germany; 3 Institut für Geographie und Geologie, Universität Greifswald, Greifswald, Germany; University of Maryland at College Park, UNITED STATES

## Abstract

Gridded population data is widely used to map fine scale population patterns and dynamics to understand associated human-environmental processes for global change research, disaster risk assessment and other domains. This study mapped gridded population across Germany using weighting layers from building density, building height (both from previous studies) and building type datasets, all created from freely available, temporally and globally consistent Copernicus Sentinel-1 and Sentinel-2 data. We first produced and validated a nation-wide dataset of predominant residential and non-residential building types. We then examined the impact of different weighting layers from density, type and height on top-down dasymetric mapping quality across scales. We finally performed a nation-wide bottom-up population estimate based on the three datasets. We found that integrating building types into dasymetric mapping is helpful at fine scale, as population is not redistributed to non-residential areas. Building density improved the overall quality of population estimates at all scales compared to using a binary building layer. Most importantly, we found that the combined use of density and height, i.e. volume, considerably increased mapping quality in general and with regard to regional discrepancy by largely eliminating systematic underestimation in dense agglomerations and overestimation in rural areas. We also found that building density, type and volume, together with living floor area per capita, are suitable to produce accurate large-area bottom-up population estimates.

## Introduction

Within the last decades, global population increased rapidly. While 3.0 billion people lived on Earth in 1960, this is anticipated to reach approximately 10.0 billion by 2060 [1]. The regional and local dynamics of this global growth are highly diverse and can be traced back to a complex interplay of factors such as economic development and restructuring, urbanization and mobility, social, cultural and political frameworks, medical capacities, conflicts or climate change related effects that all affect either fertility and mortality rates or migration [2]. Data about patterns of human population are essential to understand the relation between those factors and underlying societal or human-environmental processes and are key requirements for international development frameworks such as the Sustainable Development Goals [3] or the United Nations Paris Agreement [4]. Population is a key variable in socio-economic metabolism and sustainability pathway research [5], and population density has recently been proposed as Essential Societal Variable [6].

As census data is limited to administrative census units, gridded population approaches are a popular alternative [7]. These redistribute national or regional census population counts to smaller, grid-cell based target units that better represent local population patterns. Gridded population has been used to inform research in several domains, such as climate change and related health research [8–10], quantification of ecosystem services [11], urban development and urbanization patterns [12, 13], disaster risk assessment and exposure [14, 15] or settlement characterization and categorization [16, 17]. To date, a wide range of freely accessible global products with varying spatial and temporal resolution have been developed, including the Global Rural-Urban Mapping Project (GRUMPv1, [18, 19]), Gridded Population of the World (GPWv4.11, [20]), the Global Human Settlement Population Layer (GHS-POP, [21, 22]), LandScan [23, 24] or WorldPop ([25–27]). An overview including global and continental products and their specifications can be found in Ref. [7]).

The redistribution of national census population to smaller grid cells is commonly based on a weighting layer. In an *area-weighted* approach, population is equally redistributed to all land-surface cells, e.g. in GPWv4 [28]. *Dasymetric mapping* techniques refine this procedure by incorporating spatial ancillary data that is presumably related to population presence and density and that affects redistribution weights of each individual cell [29, 30]. *Binary* dasymetric approaches redistribute population using one or more ancillary layers that describe presence or absence of populated areas. This can include mask layers such as protected areas, steep slopes or non-built-up land cover types (e.g. in [31]), where population is not expected (*limited* variables, [32]) or data that identify built-up cells that population can be redistributed to. *Weighted* dasymetric approaches account for one or more ancillary datasets assuming that they are unequally related to population density (*related* variables, [32]) and population is redistributed based on a cell-based weight. Weights can directly correspond to ancillary features (e.g. road density can regionally relate directly to population density [33]) or can be derived from ancillary layers with an unknown a-priori relation to population based on modelling. A common way is random forest (RF) modelling, where population density or a weighting response variable is predicted using ancillary data such as land cover information, night-time lights, climatic data, topographic information or vector-based features [26]. This procedure identifies previously unknown relations and can outperform binary dasymetric mapping [31] and are, for example, used in contributions to WorldPop [26]. Hybrid approaches combine a modelled weighting layer within a previously masked settlement area [34].

Settlement layers are a key information to map gridded population [34, 35]. Over the last years, important advances have been made with regard to mapping settlements on the national to global scale from remote sensing, improving spatial and temporal resolution as well as accuracy [36]. The Global Human Settlement Layer (GHSL, [37]) and the Global Urban Footprint (GUF, [38]), continued by the World Settlement Footprint (WSF, [39]), are among the best-known global products. Research showed that a continuous representation of settlements through density maps is advantageous to further refine gridded population maps [33, 40–42], because a densely built-up area can potentially house more people than a same-sized, but less densely built-up area. Currently, however, GHS-POP is the only approach that globally accounts for settlement density, deriving density at 250m spatial resolution from an averaged high-resolution binary layer.

Despite these advances and the considerable accomplishments of globally redistributing census population to a grid-cell level, current state-of-the-art research faces four major challenges:

- **Building type:** Particularly, *de jure* approaches that redistribute population based on the legal place of residence, currently used in most products [7], require a fine representation of where people can permanently live. Even though many global gridded population maps do account for settlements, they do not account for the respective building type. This is, however, essential because population and settlement presence correlate differently in single-family and multi-family areas and do not correlate in industrial and commercial areas. So far, building types have been incorporated only implicitly into population mapping, for example through land use data for covariate modeling [26], or in very local settings [43].
- **Building height:** Vertical building structure is a highly relevant descriptor of settlement structure in general [36, 44] and population in particular [45, 46]. As operationalized building height products are only emerging recently, it has not yet been integrated into large-area gridded population maps, even though population is also vertically distributed.
- **Census data:** Redistributing census population relies on accurate census data in the first place. Dasymetric mapping is impossible when the total population is unknown and difficult when its estimation is outdated or incomplete [7]. The quality, consistency and temporal resolution of census data varies across countries and census data might erroneously be considered accurate [32]. This is particularly important as census information is often used to both redistribute population and to validate the estimation at a finer scale (e.g. [31, 34, 47]), probably resulting in an overestimation of accuracy.
- **Data consistency and modeling:** The use of modeled weighting layers based on ancillary data can introduce uncertainty based on variations in local data quality. Furthermore, physical relations between population and ancillary data are hard to quantify when weighting layers are derived from RF modeling [7]. Those statistical relations might also be regionally specific and the resolution of available census data can be important when transferring models to regions where they are different [48].

As there is a demand for gridded population data in places where administrative census units are large or in countries with quickly increasing population, high migration rates and less frequent or accurate census [7], bottom-up approaches are promising, since they do not require spatially exhaustive census data to estimate gridded population for the entire region. They seem, thus, potentially more robust towards spatially incomplete data or if national census data is outdated. However, bottom-up approaches are rather rare as they rely on detailed survey information or other high-resolution data. This is why, despite the lower cost compared to national census, bottom-up mapping has focused on settings with good data coverage so far [49]. To date, bottom-up population estimates mainly focus on specific demographic phenomena [50] and local to sub-national analyses only [43].

The goal of this study was to contribute to an accurate, large area and fine-scale gridded *de jure* population estimate using both census-based top-down dasymetric mapping and a bottom-up approach on a nation-wide scale. We established a workflow that derives population estimates for the year 2018 on a grid cell level and that responds to the identified challenges of current large-area products. We used three covariate layers that provide a direct physical relationship to population without statistical modelling. All layers were derived at 10 x 10 m^2^ spatial resolution from freely available, temporally and globally consistent Copernicus Sentinel-1 A/B and Sentinel-2 A/B (from here S1 and S2) imagery as well as OpenStreetMap (OSM) data, thus, minimising the use of region-specific ancillary covariates. We incorporated (1) a building density layer that quantifies the share of building-covered surface, (2) a building height layer and (3) a layer that indicates the type of buildings including major residential and non-residential types. Those layers were used as an input to both binary and weighted dasymetric mapping as well as the bottom-up approach using literature-guided calculations on living floor area per capita. Our study area was Germany as a) both building density and building height layers were already available and validated for this country and b) accurate census data at different administrative levels and information about regional living conditions were available for a proof-of-concept. This study specifically addressed the following research questions:

1. How do building density, building type and building height data improve the top-down redistribution of census population at different spatial scales?
2. How accurate are gridded bottom-up population estimates based on regional living conditions using building density, building type and building height?
3. How sensitive are bottom-up estimates to spatially outdated and temporally incomplete data?

## Study area

**Fig 1:**
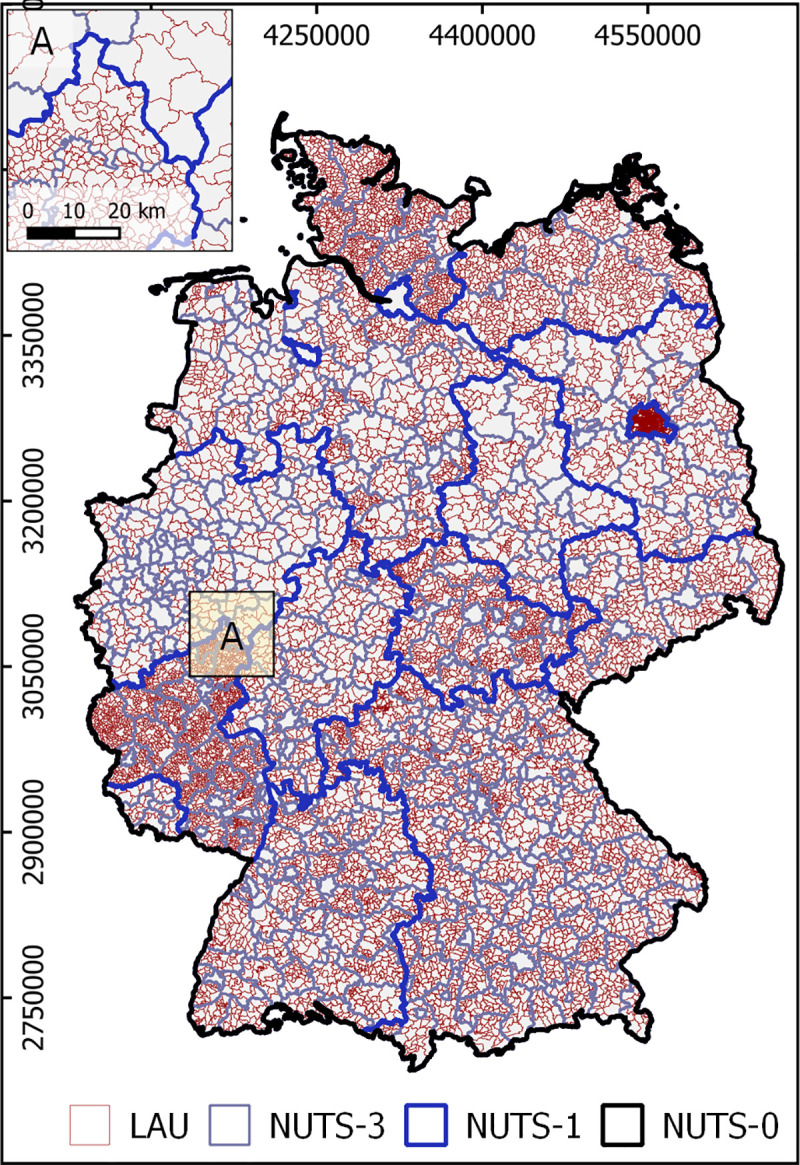
Study area administrative units. NUTS (Nomenclature des unités territoriales statistiques) and LAU (Local Administrative Units) in the study area. LAU delineations within Berlin correspond to local planning areas, Proj: ETRS-89. Inset A illustrates different higher and lower average LAU sizes across federal states. Administrative boundaries from *[51]* under dl-de/by-2-0 license (https://www.govdata.de/dl-de/by-2-0).

Our study area is Germany. Germany covers an area of about 357,000 km^2^ and had about 82.79 million inhabitants in 2018 [51], with a population density of about 232 inhabitants km^-2^. Settlement structure in Germany is highly diverse and characterized by both dense agglomerations and rural structures, with population density ranging from 69 km^-2^ to 4,055 km^-2^ in its federal states [51] and large urban-rural gradients. This study uses administrative areas from Eurostat NUTS (*Nomenclature des unités territoriales statistiques*) and LAU (Local Administrative Unit) territorial units [52] as well as Planning Areas for the city of Berlin [53]. Germany (as a NUTS-0 unit) has 16 Federal States (among which Berlin, Hamburg and Bremen are city-states) on a NUTS-1 level, 401 districts on a NUTS-3 level and 11,267 municipalities on a LAU level. The city of Berlin counts 448 planning areas (BPA, Fig 1). The average spatial resolution (ASR), a widely used measure in gridded population mapping and defined as the square root of area divided by the number of administrative units [54, 55], is 149.50 km for NUTS-1 units, 29.86 km for NUTS-3 units, 5.63 km for municipalities, and 1.41 km for BPA. We did not consider 38 NUTS-2 level regions, as their scale is similar to NUTS-1 units in some (mostly eastern) and similar to NUTS-3 units in other (mostly western) parts of Germany.

## Materials and methods

This study mapped gridded population using three covariate layers derived from S1 and S2 A/B time series, as well as from crowd-sourced OSM data. (1) We generated a building density layer based on a previously established workflow [56] and used (2) a previously generated building height layer [57]. (3) A building type layer was specifically developed for this study. Census population is used as an input to population redistribution and for validation on different scales in the top-down approach, as well as to parameterize the bottom-up approach. A list of acronyms specific to this study or to gridded population mapping can be found for reference in Table 1.

**Table 1:** List of acronyms.

Acronym	Full form
NUTS-*	Nomenclature des unités territoriales statistiques, EUROSTAT statistical administrative units
LAU	Local Area Units, EUROSTAT municipal administrative units
BPA	Berlin Planning Areas
BD-BUILD	Binary Dasymetric Mapping with Building Presence
BD-RESI	Binary Dasymetric Mapping with Residential Building Presence
WD-DENS	Weighted Dasymetric Mapping with Building Density
WD-VOL	Weighted Dasymetric Mapping with Building Volume
WD-VOLADJ	Weighted Dasymetric Mapping with Adjusted Building Volume
BU-LFA	Bottom-up Mapping using Living Floor Area
MAPE	Mean Absolute Percentage Error
REE	Relative Estimation Error
LFA/cap	Living Floor Area per Capita
IC/MF/SF/LS	Building Types: Industrial & Commercial, Multi-Family, Single-Family Buildings, Lightweight Structures
ASR	Average Spatial Resolution
RMSE	Root Mean Square Error

### Census data

Population of the study area in 2018 (reference date: 31 Dec 2017) was retrieved from the German Federal Agency for Cartography and Geodesy [51]. Those data contain official population counts on a national level (NUTS-0) as well as for all NUTS-1, NUTS-3 and LAU units. Population counts are based on continuous updates of the 2011 census [58] and account for natural population change (births and deaths) and net migration (immigration, emigration and sub-national migration, [59]). Actual census data from 2011 was used for calibrating the bottom-up estimate. Population counts for BPA (reference date: 01 Jan 2019) were retrieved from the Berlin Senate Department for Urban Development and Housing [53].

### Earth Observation-based data

#### Building density

**Fig 2:**
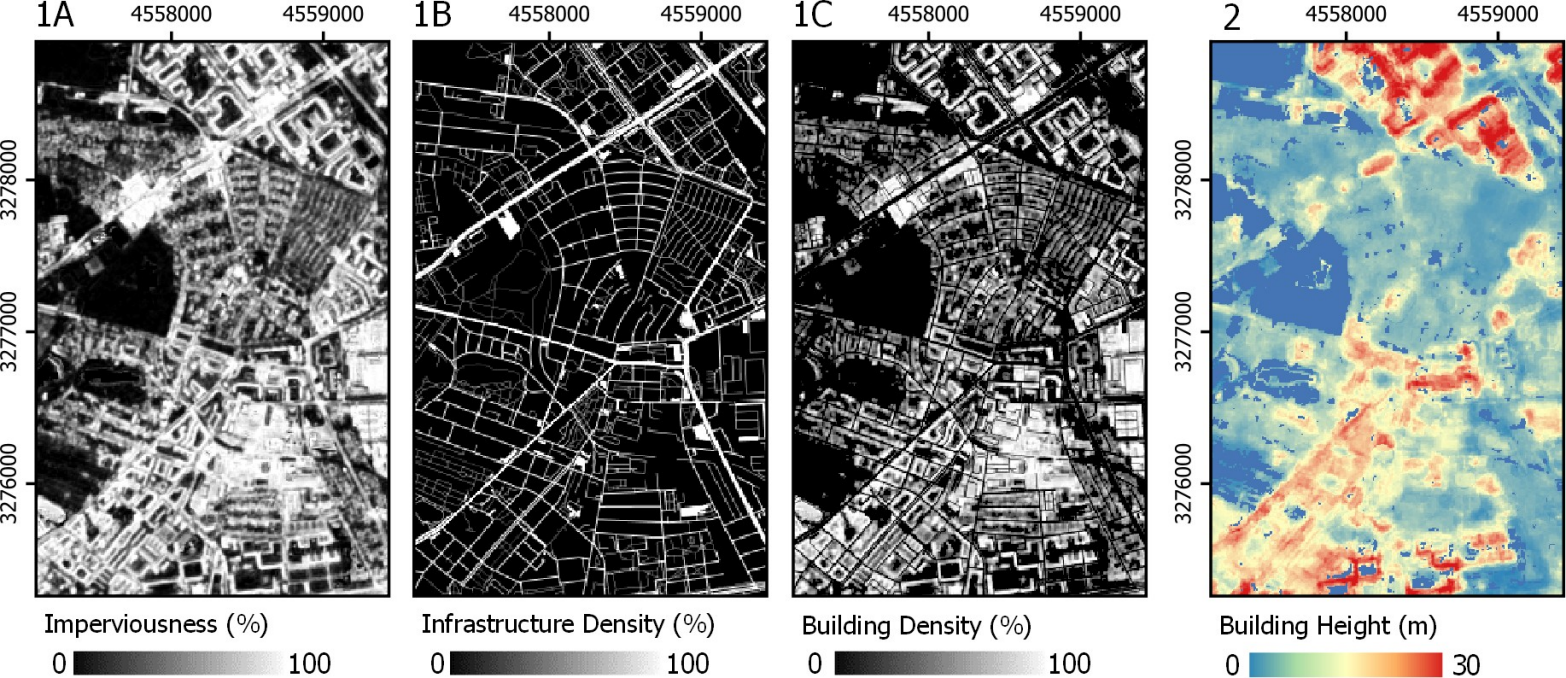
Earth Observation-based covariate layers. (1A) Imperviousness and (1B) infrastructure density from OSM to generate (1C) building density. (2) Building Height. Projection: ETRS-89.

We generated a building density layer derived from a S1 and S2 image time series from 2017 and 2018 that quantifies the sub-pixel share of impervious surfaces in percent with a spatial resolution of 10 x 10 m^2^ = 100 m^2^; thus, each percent equals 1 m^2^ of impervious surfaces (Fig 2–1A, [60]). This imperviousness layer is based on a workflow presented and validated in [56] that used a regression-based unmixing approach and spectral-temporal metrics of S1 and S2 time series. We here expanded on this workflow with an adapted feature set (see SI 1A in S1 File). As population should only be allocated to buildings, we also used crowd-sourced OSM vector data [61, licensed under CC BY-SA 2.0, www.openstreetmap.org/copyright] to support the distinction of buildings from other impervious surfaces (Fig 2–1B, see SI 1B in S1 File). Shares of infrastructure were subtracted from shares of impervious surfaces to yield building density (Fig 2–1C).

#### Building height

This study made use of a building height layer with a resolution of 10 x 10 m^2^ (Fig 2–2 57). This layer represents the average building height within a 50 m radius. It was generated based on spatial spectral-temporal metrics of S1 and S2 time series. Those represent textural information, not only accounting for reflectance and backscatter values of a single grid cell value, but also of the surrounding cells through the use of morphological operators. A radius of 50 m was used to generate spatial spectral-temporal metrics, based on the assumption that roughness and seasonal shadow effects within that area are robust descriptors of building height [62]. In contrast to the original dataset, building height in this study was not masked with an existing settlement layer.

#### Building type classification

We generated information about building types at a spatial resolution of 10 x 10 m^2^ for all cells with a building density > 25 percent. We classified four different building types–industrial and commercial (IC), multi-family residential (MF), single-family residential (SF) and lightweight structure (LS) buildings with a random forest classification approach. IC structures relate to buildings in industrial or business parks, retail or whole sale commercial centres, but also to other non-residential buildings, such as stadiums, schools or airports. MF and SF are residential building types mainly distinguished based on their size, as SF buildings are usually smaller. LS buildings are light structures such as sheds in allotment gardens or semi-permanent mobile homes. The workflow of building type mapping and its rationale can be found in SI 2 in S1 File. We achieved an overall classification accuracy of 81.40 percent.

### Top-down redistribution of census population

**Fig 3:**
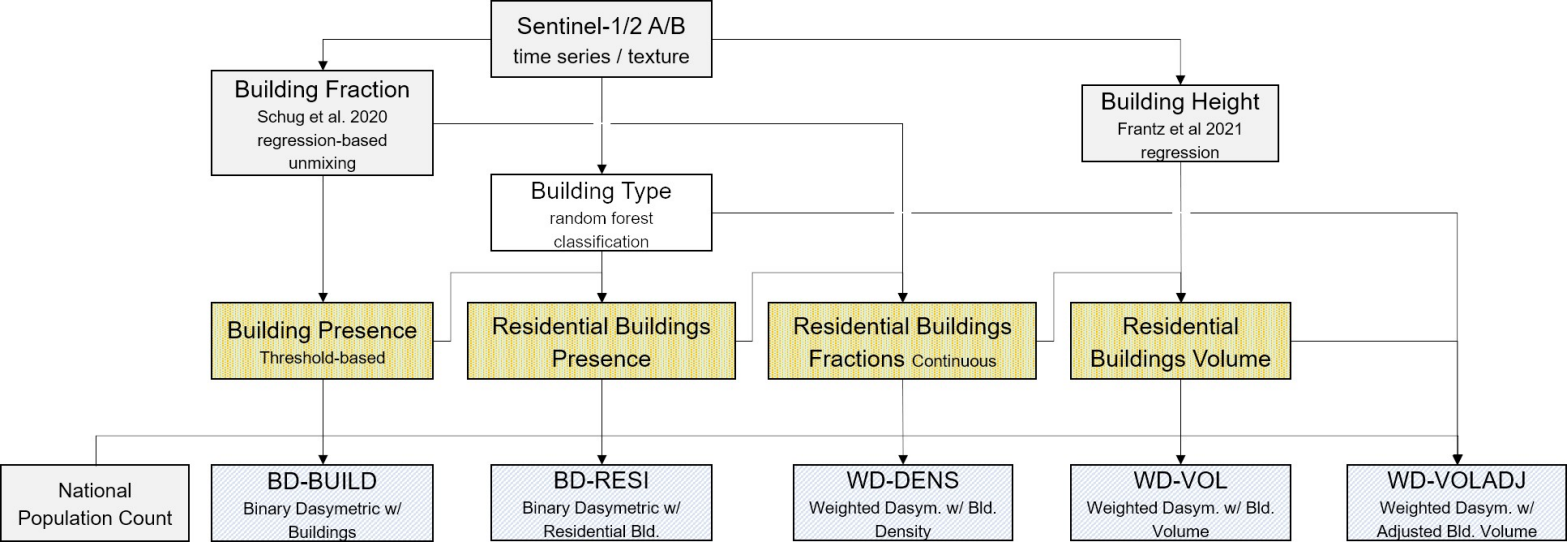
Workflow: Top-down redistribution of census population. Workflow of the data-driven redistribution of census data using different dasymetric mapping approaches. Refer to text for details about BD-BUILD, BD-RESI, WD-DENS, WD-VOL, WD-VOLADJ.

Top-down dasymetric mapping approaches redistribute known census population to smaller scale grid-cells. We generated several gridded population maps using a gradient of increasingly complex covariate layers for redistributing NUTS-0 population from 2018 to the 10 x 10 m^2^ grid-cells (Fig 3). All covariate layers were previously described building density, height and type or their combinations.

(a) In a first step, we equally redistributed NUTS-0 population to all grid cells that contain a building (i.e. building density > 25% after the application of the correction factor). In this binary dasymetric (BD) mapping approach, all cells hold an equal amount of population. This approach is referred to as BD-BUILD. Binary dasymetric approaches are based on Eq (1), with *pop*_*i*,*j*_ being the population within a grid cell i,j, *inh*_*i*,*j*_ being the binary factor of whether a cell can be inhabited in the current model and *pop*_*total*_ being the total NUTS-0 population.

$${pop}_{i,j}={inh}_{i,j}*\frac{{pop}_{total}}{\sum_{n=0}^{i*j}{inh}_{n}}$$ (1)

(b) In a second step, this procedure was refined by equally redistributing NUTS-0 population to all building cells classified as residential area. This refinement accounted for the fact that *de jure* population can usually not be found in non-residential areas. This approach is referred to as BD-RESI.

(c) While discrete land cover classification implies that a land cover feature (here: a building) is either present or absent, settlements are highly heterogeneous areas characterized by many objects smaller than the grid cell size or objects split by multiple cells. Thus, we performed a weighted dasymetric (WD) mapping approach using building density as a weight for redistributing NUTS-0 population to residential building cells. This approach is referred to as WD-DENS. All following weighted dasymetric approaches are based on Eq (2), with *w*_*i*,*j*_ being the cell weight.

$${pop}_{i,j}=w_{i,j}*\frac{{pop}_{total}}{\sum_{n=0}^{i*j}w_{n}}$$ (2)

(d) In order to account for vertical structure, building density information was multiplied with building height to obtain cell-based building volume. Here, we use building volume as a weighting factor *w*_*i*,*j*_ to redistribute NUTS-0 population to residential building cells. This approach is referred to as WD-VOL.

(e) Using building volume for grid-based dasymetric mapping implies that an equal proportion of building area and height can be assigned to each citizen. However, there is a particular difference of living floor area per capita in SF and MF housing and previous research found that incorporating those particularities is beneficial for an accurate population estimate on a local level [63]. We here performed an empirical sensitivity analysis to adjust volume weights of MF residential housing (see SI 6 in S1 File) and adapt the weighting factor *w*_*i*,*j*_. This approach is referred to as WD-VOLADJ.

A *best product* was eventually created using the approach that provided best results. For this best product we used municipal instead of national data and redistributed it to grid-cells. This product provides the best absolute results and should be used in further analyses or applications.

### Bottom-up population estimates

**Fig 4:**
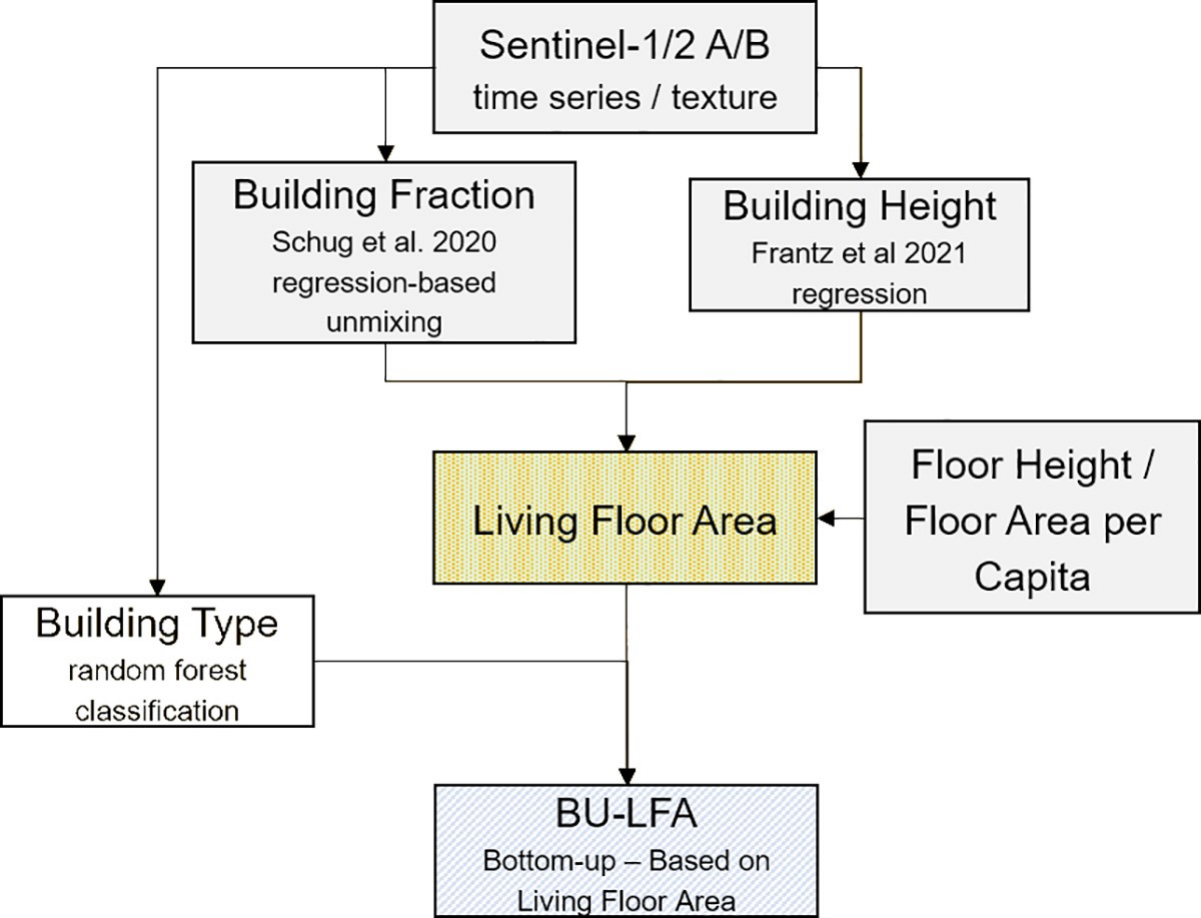
Workflow: Bottom-up gridded population. Building fraction, building height and floor area per capita for bottom-up mapping. Refer to text for details about BU-LFA.

In this bottom-up approach, we generated gridded population estimates without a-priori knowledge of the total population count. We used building density, height and type layers that are all physically related to population to compute a dataset of residential living floor area (Fig 4). Using living floor area per capita statistics, we then calculated the number of inhabitants per 10m x 10m grid-cell.

First, building height was used to derive the number of floors of SF and MF buildings per cell. We reduced building height by 3.00 m to account for roof area where population is not expected. Buildings lower than 2.00 m were excluded. We assumed a floor height of 4.50 m, based on empirical work in [63], who found a floor height between 4.00 m and 5.55 m for different building types in a local setting in South-West Germany. The number of floors was the building height divided by floor height (as decimal number, but at least 1.00). Living floor area was the number of floors multiplied with building area per cell, using an adjustment factor of 0.8 that accounted for uninhabitable areas such as walls or staircases [63]. Finally, living floor area per cell was divided by the average living floor area per capita (LFA/cap), resulting in the number of people per cell. LFA/cap was derived from census-based data on a NUTS-1 level providing the number of dwellings in SF and MF buildings and the average living floor area per dwelling, assuming that household sizes are the same (see SI 5 in S1 File). This approach is referred to as BU-LFA. As the nature of bottom-up modelling does not enforce an a-priori total, this also allows for a more independent validation compared to top-down population redistribution.

$${pop}_{i,j}=\frac{\frac{{bHeight}_{i,j}-{rHeight}_{i,j}}{{fHeight}_{i,j}}*{bResDens}_{i,j}*0.8}{{LFA/cap}_{i,j,t,s}}$$ (3)

Cell values were based on Eq (3), with *bHeight*_*i*,*j*_ being the building height in cell i,j, *rHeight*_*i*,*j*_ being the roof height, *fHeight*_*i*,*j*_, being the floor height, *bResDens*_*i*,*j*_ being the density of residential buildings in a cell and *LFA/cap*_*i*,*j*_,_*t*,*s*_ being the living floor area per capita of a cell with building type *t* in state *s*.

### Spatial and temporal bottom-up mapping sensitivity

Finally, we mapped gridded population using a bottom-up approach based on building density, type and height, assuming that LFA/cap information is spatially incomplete or temporally outdated. In a first step, we calculated gridded population using yearly LFA/cap data from 1994 to 2017 and compared the outcomes to a model using data from 2018, all based on averaged LFA/cap value from all NUTS-1 areas. In a second step, we calculated gridded population across the whole study area using LFA/cap information from one NUTS-1 unit respectively and compared each model to the regionalized model. This second comparison was using LFA/cap information from 2011, as updated regional data is not available for 2018.

### Quality assessment

A quantitative quality assessment of gridded population was performed using the established procedure of comparing aggregated gridded population to census reference data at fine spatial scale [e.g. 26, 34, 42]. Previous research found that the ASR ratio of validation units and the census units providing input population has a great impact on quality. A small ratio, i.e. a small offset in spatial scale, generally lead to better results as the compared units are less heterogeneous [7, 55, 64]. For the top-down approach we compared gridded population to census data on NUTS-1, NUTS-3 and LAU level. We focused on the smallest available reference unit, which represents the largest possible national-level ASR ratio between input and validation units. Bottom-up results were validated at all scales including NUTS-0. We derived model metrics of uncertainty (root mean squared error RMSE and mean absolute error MAE), coefficient of determination R^2^ and model slope.

We additionally computed relative quality metrics. These included the mean absolute percentage error (MAPE) that computes the average absolute percentage error across all validation units at the respective scale, and the relative estimation error (REE) that describes the mean error of population reference and estimate in each unit relative to its reference population (e.g. [42]). Models were also evaluated regarding REE in relation to actual census population density and spatial resolution in order to examine if additional covariate layers can equally improve population estimates in both smaller and larger areas with both high and low population density. For this purpose, we also analysed the distribution of LAU validation units and population counts within different ranges of REE (0–10%, 10–25%, 25–50%, 50–100%, > 100% over- and underestimation).

In order to further evaluate the mapping results, the *best product* dataset was visually compared to mapping results to two existing global example products—WorldPop and GHS-POP. No quantitative comparison was performed because most global datasets redistribute census data from the smallest available administrative unit to grid-cells. This way, the most accurate product possible can be provided, but no finer-scale units can provide census data for validation.

## Results

### Top-down redistribution of census population

**Fig 5:**
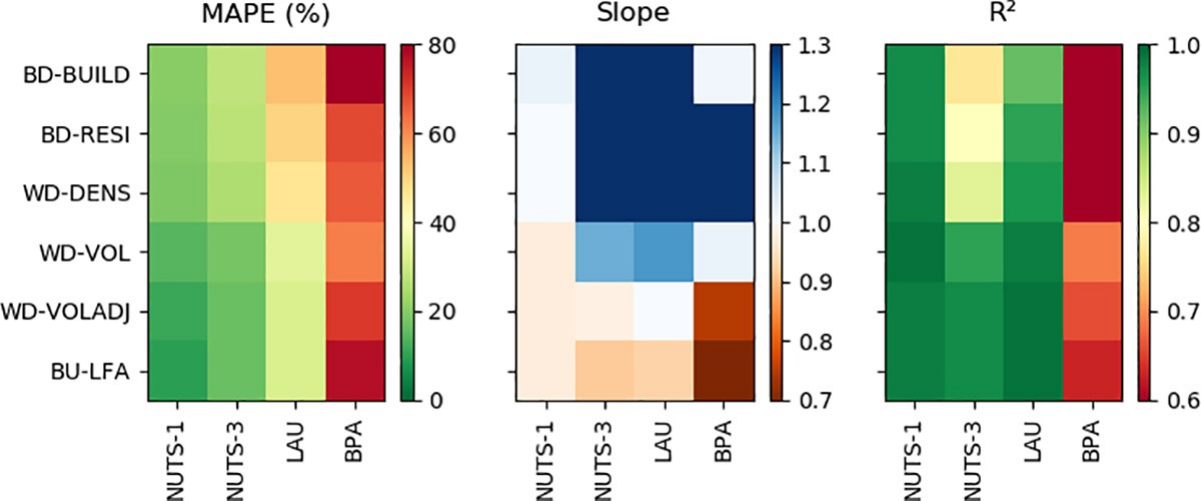
Top-down and bottom-up gridded population mapping quality. Mean absolute percentage error (MAPE), Slope and R^2^ of redistribution models at different spatial validation scales (NUTS-1 to LAU and BPA). All numbers used to create this figure in SI 8 in S1 File.

Population results can be compared along two dimensions–the applied (re-)distribution method (Fig 5, rows) and the scale of validation (Fig 5, columns). We found that in dasymetric mapping approaches (BD-BUILD to WD-VOLADJ) overall results improved along both dimensions. At all validation scales, limiting built-up areas to residential building types only is preferable, while improvements were larger on a finer validation scale. For example, MAPE decreased from 152.73% to 68.64% at BPA level, from 53.08% to 49.50% at LAU level and from 20.45% to 19.73% on a NUTS-1 level. Similar improvements could be observed for MAE, RMSE, R^2^ and Slope. Using building density, quality metrics improved slightly across all scales. The level of model quality increased markedly when building height was introduced. MAE decreased from 4,100 to 2,600 at BAP-level, from 2,100 to 1,400 at LAU-level and from 52,700 to 34,600 at NUTS-3-level. MAPE, RMSE, Slope and R^2^ improved accordingly. At a NUTS-1-level, the increase in quality was less pronounced, but still visible regarding MAPE, MAE or RMSE. Including an adjusted building volume, quality metrics improved at a NUTS-1- and NUTS-3-level, while stagnating or slightly declining at LAU- and BAP-level. We here present the results for an approach that adjusts volume weights of MF residential housing by a factor of 1.6, based on a comparison of different weighting factors. Using binary covariate layers, NUTS-1-level results largely outmatched those at finer scale units with regard to MAPE, slope and R^2^. Upon integration of building density and volume, NUTS-3- and LAU-level quality approached that of NUTS-1 with regard to slope and R^2^. While Fig 5 provides a visual representation of major quality metrics, SI 8 in S1 File provides precise numbers and scatterplots.

**Fig 6:**
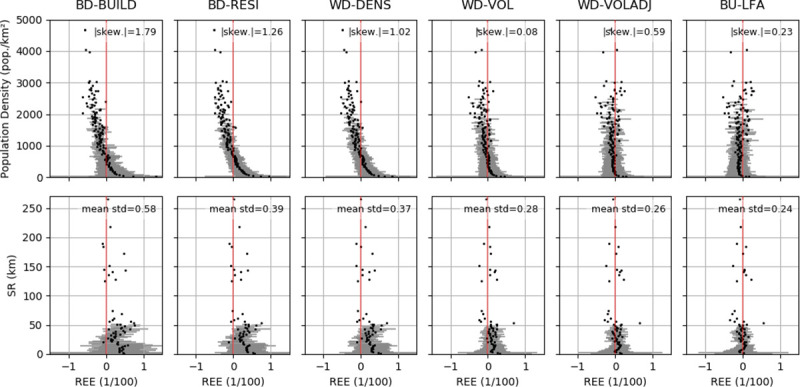
Population mapping quality in relation to population density and spatial resolution. Population density (top) and spatial resolution (bottom) of all NUTS-1, NUTS-3 and LAU areas related to their respective relative estimation error (REE) in the different redistribution models. Black dots: Mean value of y-axis bins. Grey bars: Mean values of bins +/- one standard deviation. |skew.| = absolute skewness of the means.

Using building volume reduced both the underestimation of population counts in areas with high population density and the overestimation in areas with comparatively low population density (Fig 6, top). The distribution of REE values of all validation units (NUTS-1 to LAU) showed that absolute distribution skewness decreases after integrating building density and volume, and that negative and positive REE became less related to population density. The relation of REE to spatial resolution was not as pronounced as that between REE and population density (Fig 6, bottom). However, results also improved in BD-RESI and WD-VOLADJ, where mean REE values were closer to zero and standard deviations decreased compared to the previous model.

**Fig 7:**
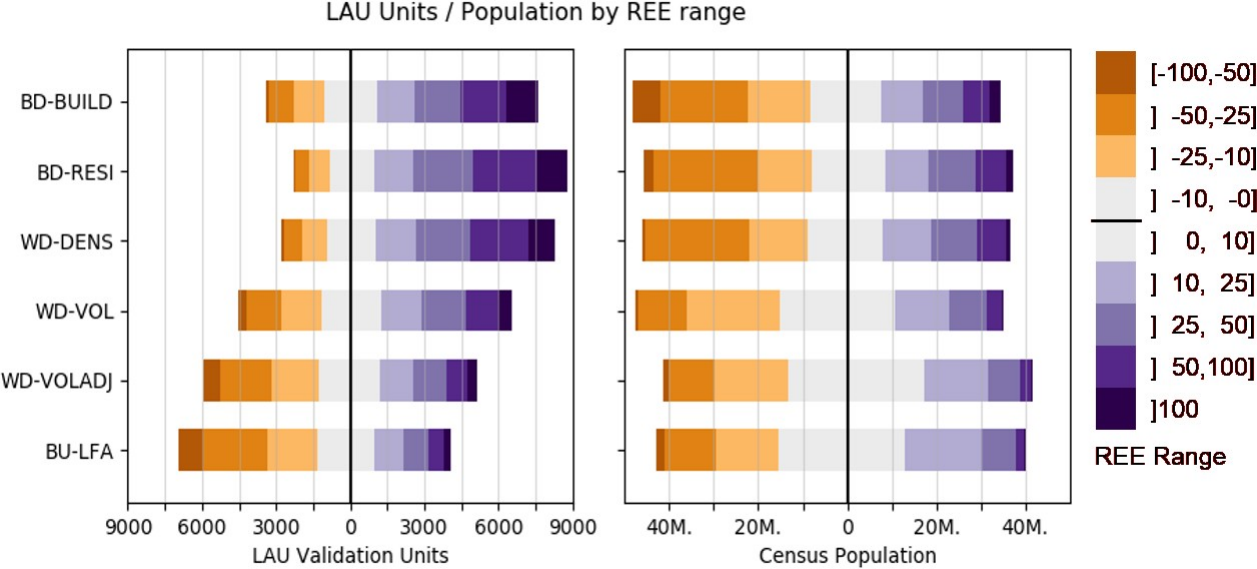
Distribution of Local Administrative Units and reference population by error range. Histogram of LAU validation units (left) and census reference population (right) within REE bins for each model. Orange colors represent underestimation, purple colors represent overestimation, grey color represents accurate predictions (REE ranges from -10% to +10%). All numbers used to create this figure in SI 7 in S1 File.

Different population models showed a different distribution of LAU (Fig 7, left) and of reference population (Fig 7, right) within REE ranges. In BD-BUILD, for example, 154 LAU units had an REE of -100 to -50%, 989 units had an REE of -50 to -25%, 1,249 units had an REE of -25 to -10%, etc. Correspondingly, for example, 8.3 million people are living in areas where BD-BUILD showed an REE of -10 to 0%. In BD-BUILD, BD-RESI and WD-DENS, only a small number of LAU showed population underestimation. In a volume-preserving approach, this resulted in a rather large number with positive REE values, as overall population estimates remain stable by definition. Results suggested that the integration of building volume helps to balance the number of LAU in which population is under- or overestimated. Compared to WD-DENS, the number of LAU with an REE between -25% and +25% increased from about 4,600 to 5,700. Similar patterns could be observed in the distribution of population. Here, the use of building volume leads to an important increase of population in areas that were modeled rather accurately, i.e. with REE values between -25 and +25% (about 58,827,000 with 25,972,000 people in areas modeled with REE between -10 and +10%) compared to using building density only (40,801,000 with 16,776,000 between -10 and +10%).

**Fig 8:**
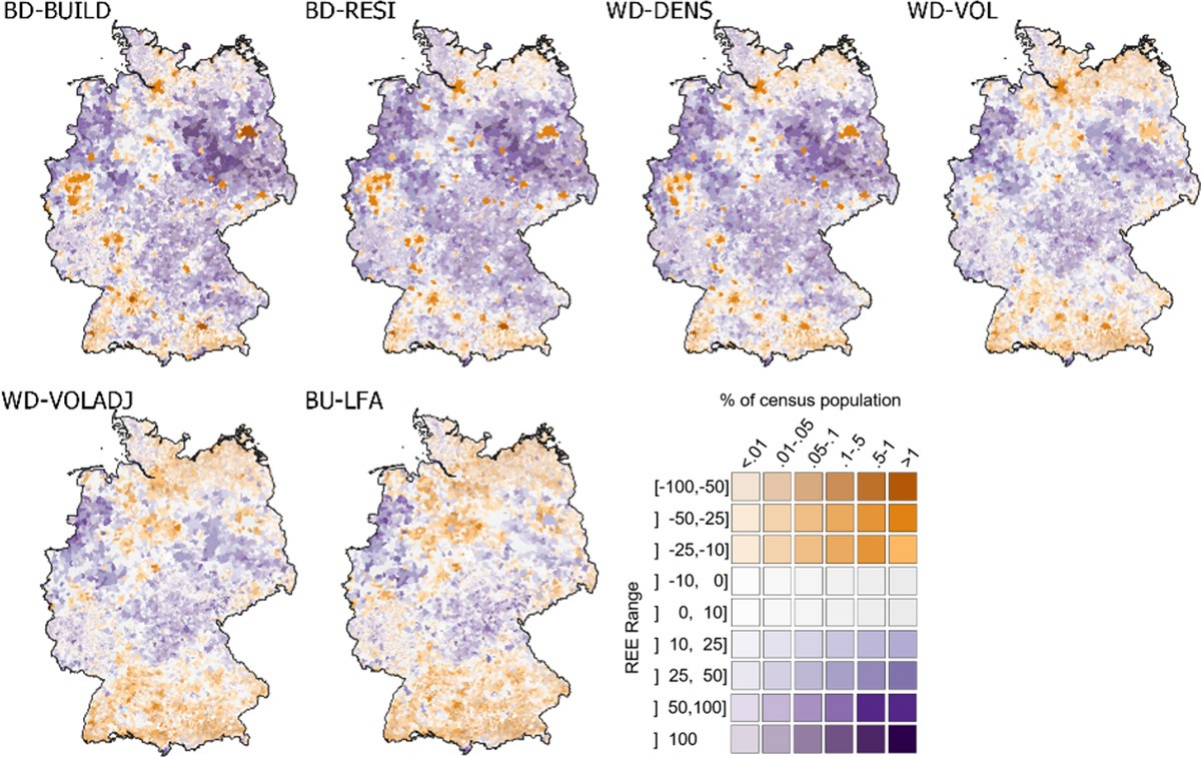
Quality of gridded population models by LAU (spatial representation). Spatial distribution of REE by LAU and model. Purple shades imply over-estimation, orange shades imply under-estimation, grey shades imply accurate predictions (REE between -10% and +10%). Administrative boundaries from [51] under dl-de/by-2-0 license (https://www.govdata.de/dl-de/by-2-0).

In BD-BUILD, BD-RESI and WD-DENS, relatively few LAU had negative REE (Fig 7). A spatial representation of REE at LAU level confirmed that population tended to be underestimated in and around agglomerations, i.e. LAU with a comparatively high population density (Fig 8). Integrating building volume in WD-VOL and WD-VOLADJ reduced differences between urban agglomerations and surrounding rural areas. Also, regional differences in REE became smaller. The number of highly over- and underestimated regions was reduced, and only few LAU remained where a large REE met high population proportions, for example in the very west of Germany.

**Fig 9:**
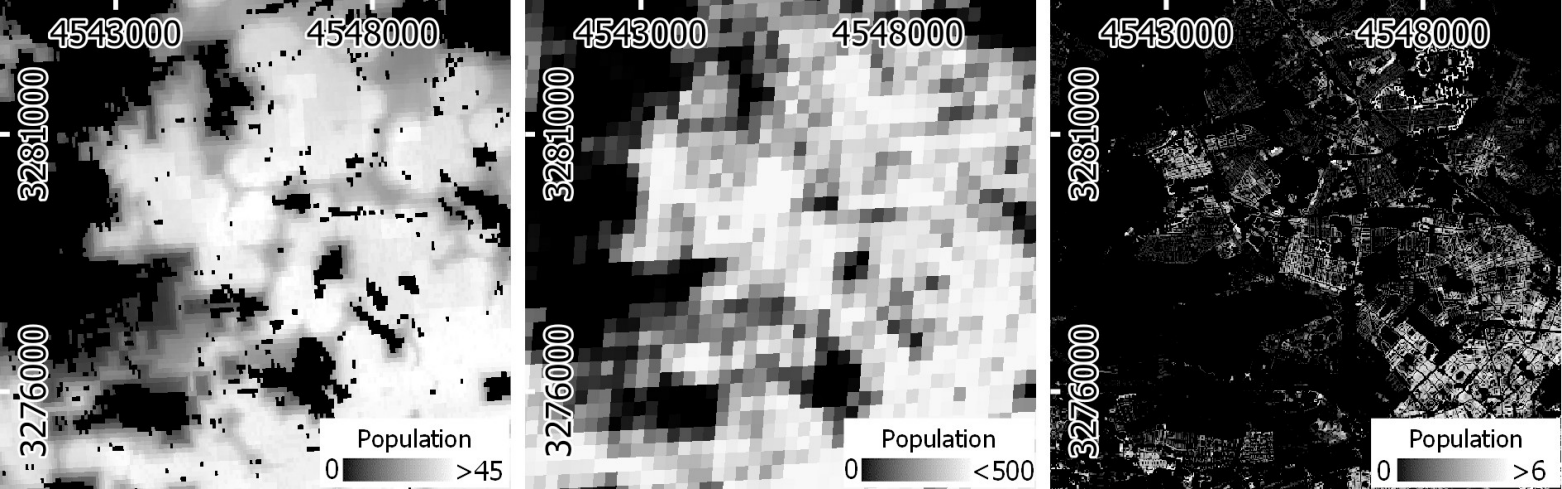
Gridded population product comparison. Comparison of WorldPop (Left, Constrained/UN-Adjusted/100m, target year 2020, [27]), GHS-POP (center, target year 2015, [21]) and the best product map from this study (right).

The *best product* map was created by redistributing municipal census reference data to 10 x 10 m^2^ grid cells using adjusted residential building volume (WD-VOLADJ). Although municipal census population is the input for all three approaches, a higher detail in population patterns can be observed in the *best product* map of this approach (Fig 9).

### Bottom-up gridded population estimates

Quality metrics of the bottom-up approach matched those of WD-VOL and WD-VOLADJ, with slightly lower MAE and RMSE at NUTS-1- and NUTS-3-level (Fig 5). This also applied to the relation of REE to actual population density and spatial resolution, with the exception of a slight underestimation of validation units with a population density of up to about 1,000 km^-^^2^, also resulting in a higher absolute distribution skewness (Fig 6). The use of BU-LFA showed a similar distribution to WD-VOLADJ with regard to the number of LAU and census population in different REE ranges, with a slightly higher number of LAU where population is underestimated (Fig 7). Accordingly, BU-LFA results were very similar to WD-VOLADJ with regard to the spatial patterns of quality, with some local particularities, for example a slightly higher population estimation in northern central Germany (Fig 8).

### Spatial and temporal bottom-up mapping sensitivity

**Fig 10:**
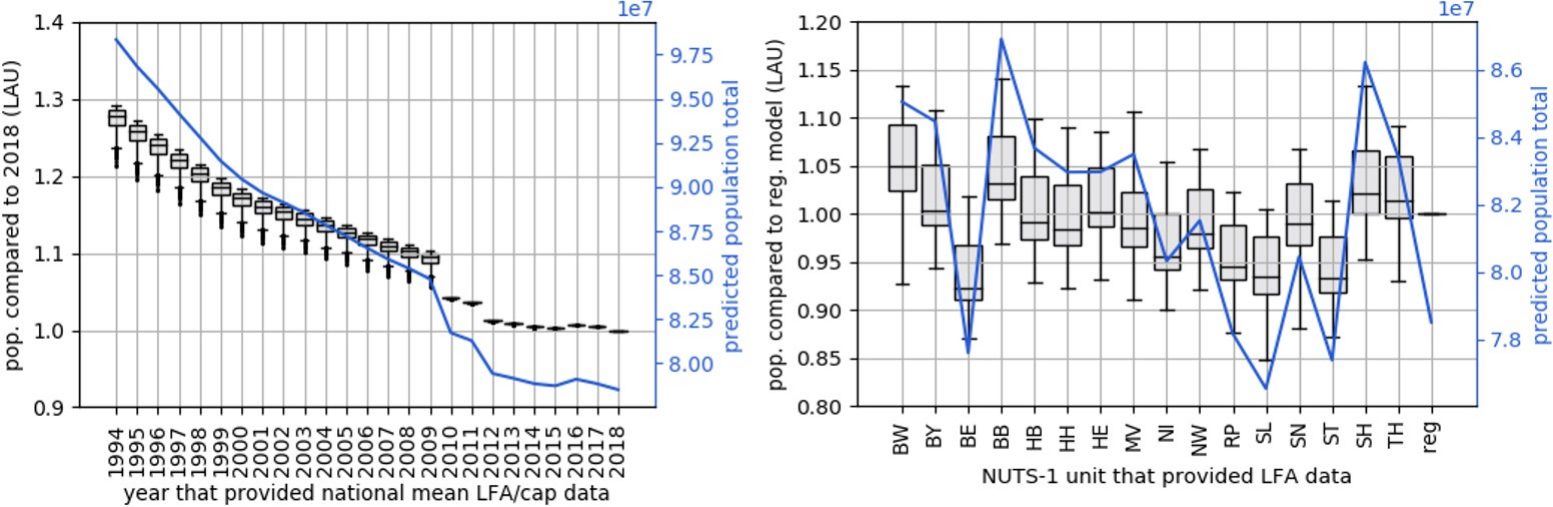
Quality of bottom-up gridded population mapping using floor area per capita from a different year or spatial subset. (Left) Ratio of population estimates within all LAU when using LFA/cap (national average) from different years compared to LFA/cap from 2018. Blue line: Predicted Population Total. (Right) Ratio of population estimates within all LAU when using LFA/cap from a single NUTS-1 unit for the whole study area only compared to individual LFA/cap per NUTS-1 unit. Blue line: Predicted Population Total.

Including LFA/cap estimates from a different spatial or temporal context in the BU-LFA models showed that using data from previous years can yield good results (Fig 10, left). LFA/cap from 2012 to 2017 did not substantially alter the estimated population at the national or municipal level. LAU population using LFA/cap data from 2012 was overestimated by about 1% compared to using LFA/cap data from 2018. Using data from 2010 and 2011, overestimations became slightly higher, with a low variance of population overestimation. Until 2009, overestimations were much higher, as smaller LFA/cap from before that year were used together with higher built-up surface density from 2018. Using LFA/cap data from 2018, overall population was estimated to be 78.84 million, as compared to a census reference of 82.8 million inhabitants.

Fig 10 (right) shows population estimates across the whole study area on a NUTS-0 (blue line) and LAU level (boxplots), assuming that LFA/cap data is only available for a spatial subset. We see that compared to the regionalized model (reg.), which used individual LFA/cap data per federal state, LAU population showed an under-/overestimation factor of 0.85 to 1.15 when using regional data only. This is also discernible in the overall population estimates, which ranged from about 74.5 million inhabitants when data from Saarland (SL) is used to about 84.9 million when data from Brandenburg (BB) is used. Using regionalized LFA/cap data from 2011, overall population is estimated to be 78.5 million.

## Discussion

### Earth Observation-based data

The quality of gridded population estimates is directly related to the availability and quality of the underlying covariate layers as well as the temporal and semantic consistency between census data and ancillary datasets. An increased amount of suitable ancillary datasets increases mapping uncertainties [7]. We here used previously established top-down dasymetric and bottom-up mapping approaches and combined them with a previously unused set of covariate layers that are directly and physically related to population.

A building density layer was created based on an established workflow to map imperviousness, but using an adapted training feature set. An extensive discussion of the workflow including possible methodological challenges can be found in the corresponding study [56]. The major challenges of using rasterized OSM data for the distinction of building and impervious non-building surfaces are discussed in the corresponding SI 1B in S1 File. An extensive discussion of the previously generated building height layer can be found in the corresponding study [62]. Current and future developments in directly mapping high-resolution building density from Earth Observation data, e.g. the convolutional neural network based approach in [65], could contribute to a more reliable distinction of building and non-building impervious surface density without additional OSM data. A building type layer was specifically created for this study. A more detailed discussion of mapping building types can be found in the corresponding SI 2 in S1 File.

### Top-down redistribution of census population

In dasymetric mapping, census data determines the initial population to be redistributed. Low quality or outdated census data bears the risk of erroneously misjudging dasymetric mapping results as redistribution and validation are based on the same dataset. We used national census data for Germany from 2018, which is based on yearly updates of the 2011 census.

BD-BUILD used a binary building layer to redistribute population. Here, population in a large number of LAU was overestimated. However, the majority of census population lived in LAU where population was underestimated and the distribution of REE related to population density showed a rather high skewness (Fig 6). The spatial representation of results confirmed that the population of urban agglomerations tended to be underestimated, resulting in an overestimation of many less populated LAU. BD-RESI excluded cells with non-residential buildings. Results improved most at finer scale, possibly because here the shares of residential and non-residential buildings are more heterogeneous across the validation units. This shows that building type information is particularly helpful to identify local population patterns. WD-DENS accounted for building density and improved overall mapping quality. This is generally in line with findings from [42]. Particularly, the share of people living in highly underestimated LAU was reduced and the REE distribution skewness decreased. This is because WD-DENS factors in higher building density in urban areas compared to lower building density in rural areas, whereas BD-RESI did not distinguish those regional particularities. WD-VOL introduced building volume as a weighting layer. Building volume largely improved quality across all validation scales, for example by reducing RMSE by about 50 percent compared to WD-DENS at NUTS-3 and LAU level. A remarkably larger portion of census population was predicted to live in areas where estimates were accurate. Volume increased model quality because population density in cells with an equal building density is further modulated through vertical building differentiation. These findings, supported by the spatial representation of results, suggest that differences in quality between densely settled agglomerations and sparsely settled areas, an issue also reported in other studies [7, 42, 47], are overcome by introducing volume. While it was shown that highly accurate 3D building models are beneficial for population mapping [45, 66], this has not yet been proven to function with potentially globally available Earth Observation products, also because those are just recently emerging [62, 67]. A volume adjustment factor further improved quality at a fine scale and the number of LAU where population is over- or underestimated became more balanced. A regionalized factor could possibly be beneficial, but would also increase the unwanted correlation of input census and validation data. Quality still showed regional patterns: For example, population in North-Western Germany, Northern Bavaria and parts of Eastern Germany tended to be overestimated, whereas population in Southern Germany and Northern Germany tended to be underestimated. In this respect, it seems that the covariate layers did not entirely represent regional heterogeneity. Nevertheless, compared to the other models, a larger share of the census population lived in accurately predicted areas. Results showed that it is generally beneficial to account for different living conditions in different building types. A quantitative comparison of the quality of our map with existing products, such as GPWv4 or GHS-POP, is challenging. As building height turned out to be a crucial element for accurate population mapping, results suggest that this approach is advantageous over products that use two-dimensional information only, such as GHS-POP or GPWv4. We also see this approach advantageous over products that require a higher modeling effort, such as WorldPop or Landscan, where the relation of covariate layers to population is a priori unknown and can be region-specific. The presented approach is also potentially applicable worldwide, as density, height and type are based on freely, globally available and consistently pre-processed data. National census data suffice for it to provide accurate high-resolution results, making it a promising approach for areas where census data is scarce. Still, the approach requires data (building height and type) that is not available globally. Future research should focus on generating such base products worldwide [36]. Also, the implementation of OSM data limits global and historic applicability due to issues of data completeness. A visual comparison of the best possible product with existing datasets showed that the use of building density, height and type is also particularly useful to map local population patterns.

### Bottom-up gridded population

In bottom-up mapping, census data provide information about local living conditions. Regional data on living floor area were only available for 2011, which could, for example, explain the underestimation of total population when regionalized data from 2011 was used in comparison to census data from spatial subsets to estimate total population (Fig 10, right). Bottom-up gridded population mapping was shown to be useful when national census data is outdated or spatially incomplete, or when living conditions across the study area are heterogeneous. However, it can still be challenging, because it requires local survey data that is relatable to the available covariate data [49]. Using layers with a direct and physical relation to population, a nation-wide bottom-up population estimate can be created with high accuracy. The quality of BU-LFA were comparable to the top-down approaches WD-VOL and WD-VOLADJ and spatial patterns were highly similar. Total population was estimated to be 78.5 million, which is an underestimation of 5.7% compared to census population. The required additional input is limited to assumptions about average LFA/cap and housing characteristics such as floor and roof height. Those are a potential source of error, as spatial and thematic granularity of input data (e.g. detail of differentiation for SF and MF housing) might be driving regional quality. Future research on bottom-up gridded population mapping should ask how reliable and representative data on living floor area per capita or similar metrics (e.g. building volume per capita [68]) can be best derived for large areas, also with regard to different socio-economic environments and generally data-scarce regions.

### Spatial scale

Scale is a particular subject of interest in gridded population mapping and can be discussed along two dimensions: 1) the minimum mapping unit and its suitability for a specific application and 2) the aggregation scale of the gridded data for validation purposes. Cell size is inherently depending on the spatial resolution of covariate layers. Here, the resolution of building density, height and type layers was 10m, as this was the native resolution of the underlying Earth Observation data. Thus, we redistributed NUTS-0 census data to a 10 x 10 m^2^ grid. This is an advantage compared to large area products commonly available at a 100m resolution or coarser, as high-resolution maps make it possible to describe local patterns with high detail. However, we suggest that gridded population results at different resolutions should be used for different purposes. As building type data was validated at a resolution of 10m, information about non-inhabited industrial or commercial areas can be reliably derived at this level. Furthermore, 10m resolution results can account for population in very small and widely scattered settlements potentially unaccounted for at 100m original mapping resolution, which is important to be in line with the UN SDG principle to leave no one behind [3]. However, high resolution results are affected by the empirical OSM correction factor. While at 10m resolution, population could be wrongly allocated to paved backyards, more reliable absolute population counts could be derived through aggregation starting at a cell size of about 100 m. This was also found to be a sufficient resolution for many applications, for example service and resource allocation [7].The validation of dasymetric approaches is not entirely independent, as population to be redistributed and gridded data usually originate from the same census. While it illustrates whether the redistribution produced accurate spatial patterns, no conclusion about actual population counts can be drawn. The most accurate gridded population maps can be produced using a small offset in scale between input providing the census population to be redistributed and validation units [42]. An indicator for this offset is the ASR ratio, the ASR relation of input and validation units. Knowing that census data might sometimes only be available on a national level, it is desirable to achieve high accuracies at high ASR ratios, which is also a sign of suitable ancillary data across heterogeneous areas (Leyk et al. 2019). It is, thus, necessary that the quality of gridded population is always related to this offset in scale, also because some quality metrics referring to absolute population (e.g. MAE) are not comparable across studies otherwise.

We validated gridded population on an aggregated NUTS-1, NUTS-3, LAU and, locally, BPA level. The ASR ratio was 106.2 at LAU level, 20.0 at NUTS-3 level and 3.9 between at NUTS-1 level. Datasets where gridded population was modeled with WD-VOLADJ and BU-LFA, as well as the *best product* redistributing LAU census data to 10m grid cells in order to create the most accurate maps possible are openly available [69, 70]) and can be explored in an interactive map viewer: https://ows.geo.hu-berlin.de/webviewer/population. We found that besides the fact that overall quality metrics increased with decreasing ASR ratio for nearly any model, the impact of enhanced covariate layers including building density, type and height on quality becomes more apparent at higher ASR ratios. While overall quality metrics across all validation units are important, it is also desirable that the quality within the individual units is acceptable for a given application. The integration of (adjusted) volume contributed considerably to decoupling REE from the size and population density of the validation unit (Fig 6).

### Spatial and temporal transferability

Temporal transferability of gridded population models becomes relevant if no census data is available for the point in time to be mapped, which may rather be the rule than the exception. As dasymetric mapping approaches are volume-preserving, model transfer is more interesting in bottom-up approaches, where the suitability of LFA/cap data from a different point in time can be tested. While always depending on local conditions, related results are an indicator for overall temporal model robustness. We found that bottom-up estimates were relatively stable when using LFA/cap from 2010 to 2018. This is because before 2010, building statistics were extrapolated from the 1987 census in West Germany and the 1995 building census in East Germany [71]. Since 2010, LFA/cap was extrapolated from the 2011 census, and some of the earlier estimates were corrected according to the new data: For example, the estimated number of SF and MF units was reduced by about 900.000 from 2009 to 2010 with an increasing total living floor area, leading to a jump in LFA/cap statistics. Population before 2010 was overestimated, because lower LFA/cap statistics at this time are now used together with higher building volume.

Spatial transferability of gridded population models becomes relevant if no area-wide census is available. We therefore tested the quality of bottom-up population estimates if LFA/cap was available from a subregion only. Note that the results considering spatial and temporal transferability are not comparable among each other, as regional LFA/cap on a NUTS-1 level could only be derived for 2011 and with a slightly different approach than historic LFA/cap (see SI 5 in S1 File). We mapped nation-wide population using LFA/cap from one selected NUTS-1 region at a time and compared the results to using a regionalized model (i.e. as in BU-LFA). Population is over- or underestimated by up to ca. 10%, depending on the region that contributed LFA/cap. Thus, results showed that data from a subregion can generally be used for bottom-up mapping as long as local living conditions are largely stable or within a fairly low margin of change, which is in line with findings of a similar study in Haiti [40]. However, the representativity of the region providing LFA/cap can have an impact on mapping results. LFA/cap from Mecklenburg-West Pomerania, featuring the lowest population density in Germany, led to a large quality range and fewer LAU were accurately mapped compared to using LFA/cap from North Rhine-Westphalia, which itself hosts nearly a fifth of the total population.

## Conclusion

We mapped gridded population across Germany to quantify how mapping quality relates to input covariate data that is directly and physically related to population. We found that an equal weighting of building cells along the urban-rural gradient and, thus, an equal distribution of population seems inadequate. While building density and building type were found to be useful for dasymetric mapping, particularly with regard to local analyses, building height improved mapping most remarkably and at all spatial scales and contributed to a more spatially equal distribution of mapping quality. Foremost, building height reduced the underestimation of population in dense urban environments and particularly increased result quality when the average spatial resolution ratio was high, which indicates that height enables accurate population maps without high-resolution census reference data.

Our approach including building height allows for creating a fine-scale picture of population distribution from both top-down and bottom-up mapping. Building height improves mapping quality across large areas and, in particular, accurately describes population heterogeneity along urban-rural gradients, i.e. in areas with both higher and lower population density. The approach is advantageous over products using two-dimensional information only or those that require an increased modeling effort. We are accordingly convinced that this study provides a valuable contribution towards more robust and accurate fine-scale gridded population mapping and suggest to make the effort to implement building type and, even though not yet consistently available across the globe, building height data into existing gridded population products. We underline the importance of providing population mapping accuracy related to the scale of validation and of using relative quality metrics. More accurate fine scale population maps will contribute to a deeper understanding of processes relevant for global and climate change mitigation, to efforts in mapping social-environmental key variables such as material stocks, as well as to the achievement of the Sustainable Development Goals, both in specific regions of interest, but also globally along urban-rural gradients.

## Supporting information

S1 File This contains information about the adaptation and creation of underlying building density, height and types data, including information about data and methodology. This material is presented in the S1 File, as we do not consider it essential for the goal of this study. **SI 1. Building density mapping** Contains supplementary information about how imperviousness maps from [56] were transformed into building density maps using rasterized OpenStreetMap data. **SI 2. Building type mapping** Contains further information about how a building type layer was modeled from Sentinel-1 and Sentinel-2 Earth Observation data using Random Forest modeling. **SI 3. Opening and closing** Contains further information about morphological metrics used to classify building types. **SI 4. Federal state abbreviations (NUTS-1 units)** Contains federal state abbreviations used in this study. **SI 5. Living floor area per capita** Contains information about how living floor area per capita values were calculated for bottom-up population estimates. **SI 6. Adjusted building volume** Contains results from the sensitivity analysis conducted to select a volume-adjusted dasymetric mapping model (e.g. presented in Fig 5). **SI 7. Validation units and census population within REE ranges** Contains numerical representation of results that were presented graphically in Fig 7. **SI 8. Gridded population results** Contains a scatterplot representation including numerical results for all gridded population mapping models as presented graphically in Fig 5.
(DOCX)
