# Supplementary material for: Gridded population mapping for Germany based on building density, height and type from Earth Observation data using census disaggregation and bottom-up estimates
Source: PLoS One. 2021 Mar 26;16(3):e0249044. doi: 10.1371/journal.pone.0249044 (PMC7996978; doi:10.1371/journal.pone.0249044)
Supplement: S1 File — This material is presented in the S1 File, as we do not consider it essential for the goal of this study. SI 1. Building density mapping Contains supplementary information about how imperviousness maps from [56] were transformed into building density maps using rasterized OpenStreetMap data. SI 2. Building type mapping Contains further information about how a building type layer was modeled from Sentinel-1 and Sentinel-2 Earth Observation data using Random Forest modeling. SI 3. Opening and closing Contains further information about morphological metrics used to classify building types. SI 4. Federal state abbreviations (NUTS-1 units) Contains federal state abbreviations used in this study. SI 5. Living floor area per capita Contains information about how living floor area per capita values were calculated for bottom-up population estimates. SI 6. Adjusted building volume Contains results from the sensitivity analysis conducted to select a volume-adjusted dasymetric mapping model (e.g. presented in Fig 5). SI 7. Validation units and census population within REE ranges Contains numerical representation of results that were presented graphically in Fig 7. SI 8. Gridded population results Contains a scatterplot representation including numerical results for all gridded population mapping models as presented graphically in Fig 5. (DOCX) [file pone.0249044.s001.docx]

**S1 File.**

**Gridded population mapping for Germany based on building density, height and type from Earth Observation data using census disaggregation and bottom-up estimates**

Franz Schug^1,2,*^; David Frantz^1^; Sebastian van der Linden^3^; Patrick Hostert^1,2^

^1^ Earth Observation Lab, Geography Department, Humboldt-Universität zu Berlin, Berlin, Germany

^2^ Integrated Research Institute on Transformations of Human-Environment Systems, Humboldt-Universität zu Berlin, Berlin, Germany

^3^ Institut für Geographie und Geologie, Universität Greifswald, Greifswald, Germany

* corresponding Author e-mail: franz.schug@geo.hu-berlin.de (FS)

## Building density mapping

## Imperviousness mapping

A workflow of mapping nation-wide sub-pixel shares of multiple land cover types was established and validated in [1]. This workflow used a machine learning regression-based spectral unmixing approach with artificially mixed training data as described in detail in 2). We here expanded on this workflow with an adapted STM feature set using the 1st, 2nd and 3rd quartile of S2 reflectance, as well as mean and standard deviation of Tasseled Cap Greenness and mean S1 VH-polarized backscatter data from all image acquisitions, as this feature set proved to be more robust for separating impervious surfaces from seasonal soil [3]. At 270 training sites for impervious surfaces across the study area, we retrieved spectral-temporal metrics (STM), i.e. pixel-based time series statistics of reflectance or backscatter [4,5]. Those were then used in the regression to quantify impervious surfaces. Validation using Google Earth © imagery led to an RSME of 19%, MAE of 13%, R² of 0.74 and model slope of 0.75.

## OpenStreetMap for building density

OSM polylines of 39 *highway* classes, 12 *railway* classes as well as polygons of *runways*, *aprons*, *taxiways* and *parking* *lots* across the study area were extracted from 6). Highway and railway data were buffered with lane and rail widths based on expert-guided estimates. Polygon and buffered polyline data were rasterized, i.e. for each 100 m² grid cell, the area share [%] covered with infrastructure was computed.

The workflow of OSM data handling including literature-based assumptions is described in detail in Haberl et al. (in review). As smaller infrastructure such as paved yards or private parking lots are often not included in OSM, we applied a correction factor of 0.57 to the remaining building density. This factor was empirically derived based on a linear regression between generated building density information with rasterized cadastre building footprints of three NUTS-1 units - the city of Berlin and the states of Thuringia and North Rhine-Westphalia (Fig. SI 1.1). As R² is increasing with decreasing resolution, we chose to derive the factor at 1000 m resolution. Note that this factor varies regionally, but its variation is rather low across the sites, which suggests a stable correlation. During processing, building density at 10 m spatial resolution was multiplied with this factor. To minimise commission error, we further excluded grid cells with a building density lower than 25 percent, which approximately coincides with the uncertainty (RMSE) of the impervious fraction mapping.


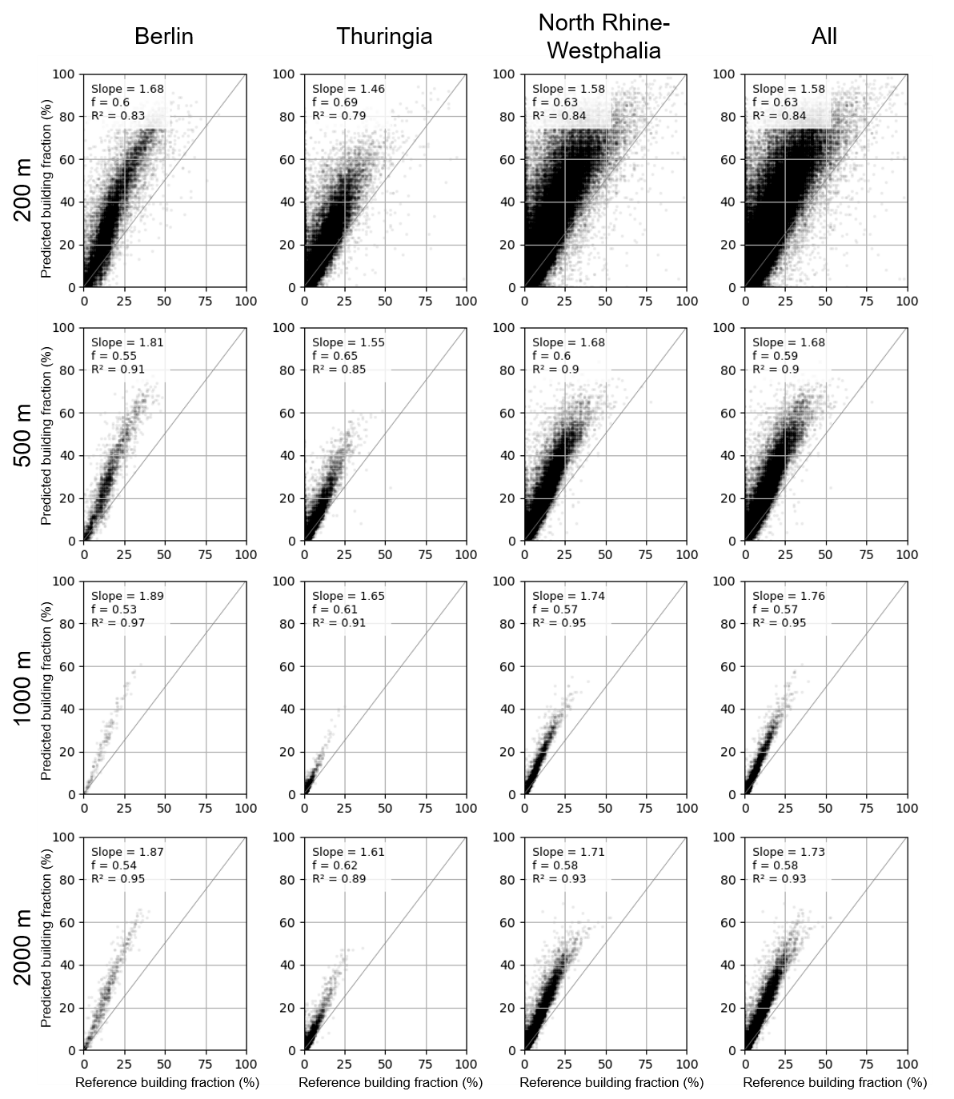


**Figure SI 1.1** Predicted building fractions after subtraction of rasterized OSM infrastructure data from the impervious fraction map related to reference building fractions from rasterized cadastre data at different spatial resolutions. A correction factor was derived based on the systematic linear overestimation of predicted building fractions.

The use of rasterized OSM data for the distinction of buildings and non-building impervious surfaces brings along three challenges: 1) OSM data is rarely providing time stamps, introducing uncertainties through a temporal gap between the data download in 2020 and the target year 2018. 2) OSM does not guarantee feature completeness, as data is crowd-sourced. 3) Literature-derived national road widths might not have been suitable to buffer each OSM line feature. We assume the impact of these issues to be minor, as 1) road network length within the study area was largely stable from 2000 to 2019 [7] and 2) an empirical correction factor was introduced to account for impervious surfaces not covered by OSM data.

## Building type mapping

We re-utilized all available analysis-ready S1 observations of 2017 and all S2 observations of 2018 with a cloud-coverage < 70% across the study area as described in 8). We generated STMs from all observations. Then, S1 and S2 STMs were used to derive 48 SSTMs using opening and closing operators of the median (Q50) and the inter-quartile range (IQR) of eight S2 reflectance bands, three spectral indices as well as median (Q50) S1 VH-polarized backscatter based on a circular kernel of 50 m radius (Table SI 2.1). Q50 and IQR have been chosen as an input to SSTM creation in order to cover both average surface conditions and variation throughout the year. Processing was done using the Framework for Operational Radiometric Correction for Environmental monitoring (FORCE, 9).

SSTMs in general have been successfully used to classify settlement types due to their ability of characterizing spatial context, for example in mapping Local Climate Zones [10]. A radius of 50 m implies that the building type can be identified based on spectral information of its immediate surroundings and is at the lower end of neighbourhood sizes commonly used to map broader neighbourhood characteristics [11,12].

**Table SI 2.1** S1 and S2 features used to derive Opening and Closing SSTMs. NDVI = Normalized Difference Vegetation Index [13], NDBI = Normalized Difference Built-up Index [14], MNDWI = Modified Normalized Difference Water Index [15]. NIR = Near Infrared, SWIR = Short-wave Infrared.

| **Features for Opening and Closing metrics** |
| --- |
| S1 VH polarized median & IQR (4 metrics) |
| S2 reflectance blue median & IQR (4) |
| S2 reflectance green median & IQR (4) |
| S2 reflectance red median & IQR (4) |
| S2 reflectance red edge 1 median & IQR (4) |
| S2 reflectance NIR median & IQR (4) |
| S2 reflectance broad NIR median & IQR (4) |
| S2 reflectance SWIR1 median & IQR (4) |
| S2 reflectance SWIR2 median & IQR (4) |
| S2 reflectance NDVI median & IQR (4) |
| S2 reflectance NDBI median & IQR (4) |
| S2 reflectance MNDWI median & IQR (4) |

We employed a two-step stratified sampling approach. First, we collected one sample per class and per district (NUTS-3), resulting in 1,604 regionally balanced training samples. Sites were selected based on a visual interpretation of the building density map and VHR imagery from Google Earth ©. Training sites were collected in cells where only one building type was apparent. If possible, the cell had to be at least partly covered by a building, i.e. building density > 25%. We extracted SSTMs at the training sites with the respective class label. An RF classification was performed, using a random subset of 70% of all samples for training and 30% of the data for validation. We used an ensemble of 500 trees with the square root of the number of all features randomly selected in each tree [16].

After obtaining first results, we performed a second sampling. This was used to compensate for large NUTS-3 units with a high population and high within-class diversity that were under-represented by the equal-number sampling. We additionally selected up to 40 sample sites per NUTS-1 unit, resulting in a new total of 2,149 sites. The selection of new sites was based on RF class probabilities and RF class margins of the first mapping iteration. We concentrated the effort of re-sampling to areas subject to high model uncertainty with an RF class probability of the winning class < 60% and an RF margin, i.e. the class probability distance from the winning to the second class, < 20%. The RF procedure was repeated with the same parameterization using the increased training data.

In order to assess building type classification quality, we established a confusion matrix including overall classification accuracy as well as class-wise user’s and producer’s accuracy [17]. We additionally compared residential building type classification results to residential building type distributions from census data on a NUTS-1 level [18]. Producer’s accuracy ranged between 71.29 percent for MF and 90.23 percent for SF buildings. User’s accuracy ranged between 77.42 percent for MF and 96.55 percent for LS buildings (Table SI 2.2).

**Table SI 2.2** Confusion matrix and respective RF class accuracy based on a 30% sub-sample of all class samples, including user’s and producer’s accuracy, overall accuracy (OA)

|  | **Reference** | | | | |  |  |
| --- | --- | --- | --- | --- | --- | --- | --- |
|  |  | **IC** | **SF** | **LS** | **MF** | **n** | **User’s Acc.** |
| **Pred** | **IC** | 159 | 4 | 2 | 16 | **181** | 87.84 % |
|  | **SF** | 10 | 194 | 3 | 42 | **249** | 77.91 % |
|  | **LS** | 0 | 1 | 28 | 0 | **29** | 96.55 % |
|  | **MF** | 26 | 16 | 0 | 144 | **186** | 77.42 % |
|  | **n** | **195** | **215** | **33** | **202** | **645** |  |
|  | **Prod. Acc.** | 81.54 % | 90.23 % | 84.48 % | 71.29 % |  | **OA = 81.40 %** |

Across the study area, about 24 percent of all classified cells were categorized as IC, 62 percent as SF, 2 percent as LS and 12 percent as MF. While shares of LS buildings were relatively stable across the federal states, the share of IC buildings ranges from 16.02 percent in Berlin to 33.94 percent in Bremen. The share of MF is highest in the three city states (up to 43.14 percent in Berlin) and lowest in states with a low built-up density (minimum 6.93 percent in Brandenburg). SF housing ranges from 35.48 percent in the city of Hamburg to 72.16 percent in Saarland. Across all 13 areal federal states (NUTS-1), the ratio of SF to MF residential buildings was over- or underestimated by an average of 2.0 percentage points compared to 2011 census data. In the three city states (Berlin, Hamburg, Bremen), the share of MF residential buildings was overestimated by 8.85 percentage points on average. Overall, the R² achieved for the building type classification is 0.93 for SF and 0.86 for MF buildings (Figure 2.1).


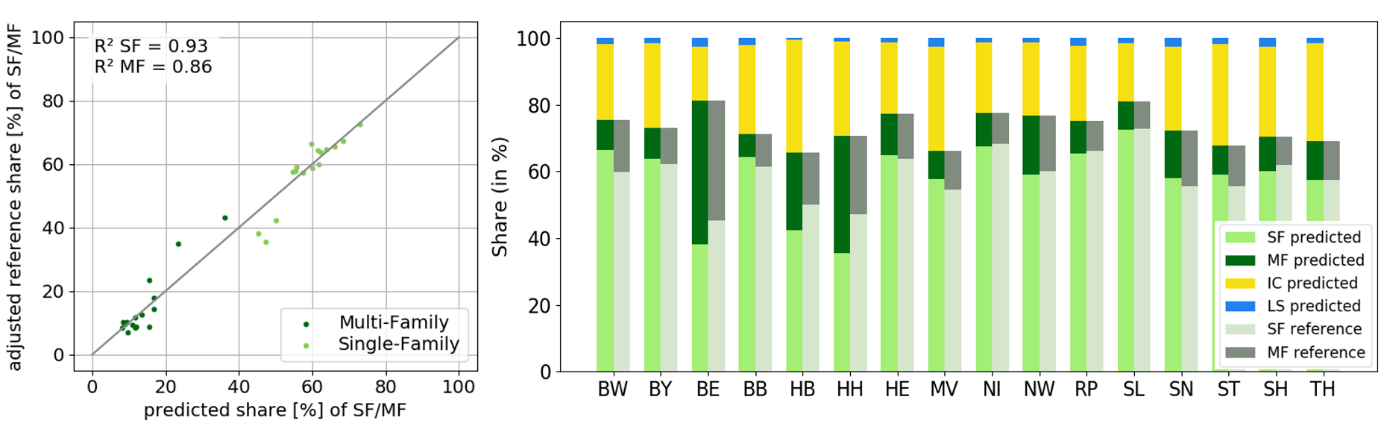


**Figure 2.1**: **Left:** Predicted share of single-family and multi-family built-up cells in all 16 NUTS-1 units compared to census references. **Right:** Relative distribution of all predicted building types (left bar) and reference residential buildings (right bar) for all 16 NUTS-1 units. State abbreviations provided in SI 3.

We mapped the type of a building suggested by its structure, as the actual building function is impossible to detect from satellite imagery (e.g. an abandoned production site now used for residential purposes). With regard to RQ1, our ability to distinguish residential (SF and MF) and non-residential (IC and LS) buildings was specifically helpful to exclude unpopulated areas. Comparing SF and MF building shares to reference data at NUTS-1 level showed that those structures were also well covered, except for a slight underestimation of SF housing in city states where the density of SF buildings is higher and more similar to MF structures than in small towns. The sampling of SF and MF housing was sometimes challenging when SF and MF buildings resembled, also complicated by the fact that SF buildings subsumed single- and two-family houses in order to be consistent with census data. In city centers, mixed use was a possible source of error and was mostly sampled as MF housing, as commercial activity usually occupies the ground floor only. Here, confusion could appear with regard to office buildings. Potential effects of the temporal gap between S1 (2017) and S2 (2018) imagery are minor, as building stocks in Germany grew by only 6‰ in 2018 [19].

## Opening and closing

Opening and closing are morphological operators based on both, in our case grayscale, erosion and dilation [20]. Opening is an erosion followed by a dilation. It is, thus, a selection of the minimum value of all pixels within the radius followed by a selection of the maximum value of those minima within the radius. Opening reduces the occurrence and size of patches with comparatively high reflectance. Closing is a dilation followed by an erosion. It is the selection of the minimum of all maxima within the SE and reduces the occurrence and size of patches with comparatively low reflectance.

## Federal state abbreviations (NUTS-1 units)

**Table SI 4** Abbreviations for German Federal States (corresponds to NUTS-1 units)

| **Abbreviation** | **Federal State** |
| --- | --- |
| BW | Baden-Württemberg |
| BY | Bavaria |
| BE | Berlin |
| BB | Brandenburg |
| HB | Bremen |
| HH | Hamburg |
| HE | Hesse |
| MV | Mecklenburg-Vorpommern |
| NI | Lower Saxony |
| NW | North Rhine-Westphalia |
| RP | Rhineland-Palatinate |
| SL | Saarland |
| SN | Saxony |
| ST | Saxony-Anhalt |
| SH | Schleswig-Holstein |
| TH | Thuringia |

## Living floor area per capita

The presented bottom-up modeling approach relied on information on living floor area per capita (LFA/cap) in both single-family (SF) and multi-family (MF) buildings. BU-LFA used regionalized data, i.e. that an individual LFA/cap for SF and MF was used for each federal state (Table SI 5.1). We derived LFA/cap_t,s_ as the ratio of the total living floor area of all units of a building type and the people living in a all buildings of that type, with *t* being the building type and *s* being the state (eq. SI 5.1). Spatial sensitivity analysis, i.e. the use of spatially incomplete LFA/cap information is conducted with data from each of the federal states. State-level census data is provided for the year 2011 [21].

(eq SI 5.1) ${LFA/cap}_{t,s}=\frac{{LFA/unit}_{t,s} * {Units}_{s} * {ShareOfUnits}_{t,s}}{{{Population}_{s}* ShareOfUnits}_{t}}$

**Table SI 5.1** Living floor area per capita (LFA/cap) for single-family (SF) and multi-family (MF) buildings in all NUTS-1 units (Federal States) and census information used in eq. SI 5.1.

| **State** | **Population** | **Units** | | **LFA/unit (m²)** | | **LFA/cap (m²)** | |
| --- | --- | --- | --- | --- | --- | --- | --- |
|  |  | **SF** | **MF** | **SF** | **MF** | **SF** | **MF** |
| BW | 11,023,425 | 2,380,413 | 2,438,236 | 116.7 | 74.8 | 51.0 | 32.7 |
| BY | 12,997,204 | 2,986,676 | 2,790,260 | 121.6 | 70.4 | 54.0 | 31.3 |
| BE | 3,613,495 | 189,026 | 1,646,180 | 115.4 | 67.3 | 58.6 | 34.2 |
| BB | 2,504,040 | 600,654 | 632,722 | 106.9 | 61.6 | 52.7 | 30.3 |
| HB | 681,032 | 116,619 | 219,460 | 111.3 | 63.6 | 54.9 | 31.4 |
| HH | 1,830,584 | 180,661 | 709,296 | 113.8 | 65.2 | 55.3 | 31.7 |
| HE | 6,243,262 | 1,398,378 | 1,409,609 | 119.6 | 72.4 | 53.8 | 32.6 |
| MV | 1,611,119 | 338,873 | 499,922 | 107.5 | 59.5 | 56.0 | 31.0 |
| NI | 7,962,775 | 2,207,784 | 1,490,347 | 122.1 | 71.0 | 56.7 | 33.0 |
| NW | 17,912,134 | 3,549,012 | 4,901,017 | 116.8 | 69.8 | 55.1 | 33.0 |
| RP | 4,073,679 | 1,166,425 | 733,289 | 122.3 | 74.2 | 57.0 | 34.6 |
| SL | 994,187 | 332,411 | 154,281 | 117.5 | 73.1 | 57.5 | 35.8 |
| SN | 4,081,308 | 953,395 | 1,284,622 | 98.7 | 62.8 | 54.1 | 34.4 |
| ST | 2,223,081 | 534,395 | 720,054 | 102.5 | 61.7 | 57.8 | 34.8 |
| SH | 2,889,821 | 742,676 | 597,895 | 115.4 | 65.4 | 53.5 | 30.3 |
| TH | 2,151,205 | 517,523 | 600,238 | 102.2 | 62.8 | 53.1 | 32.6 |

Temporal sensitivity analysis, i.e. the use of temporally outdated LFA/cap information with Earth Observation-based products from 2018, is based on historic census data [19]. Historic census data does not include NUTS-1-level information and is available on a national level including West and East Germany from 1994 on. This dataset is not entirely consistent with the 2011 census data. We derive LFA/unit for a building type using the ratio of the total area of all units and the total number of units of a type. We divide that by the average number of people living in one unit, using the ratio of the total population in that year and the total number of units, assuming that the household size in SF and MF units is comparable (eq. SI 5.2).

(eq. SI 5.2) ${LFA/cap}_{t,a}=\frac{\frac{{AreaOfUnits}_{t}}{{Units}_{t}}}{\frac{Population}{Units}}$

**Table SI 5.2** Living floor area per capita (LFA/cap) for single-family (SF) and multi-family (MF) buildings from 1994 to 2018 and census information used in eq. SI 5.2. Spatial Scale: NUTS-0.

| **Year** | **Population** | **Units** | | **Total Area (1/1000 m²)** | | **LFA/cap (m²)** | |
| --- | --- | --- | --- | --- | --- | --- | --- |
|  |  | **SF** | **MF** | **SF** | **MF** | **SF** | **MF** |
| **1994** | 81,338,000 | 15,897,356 | 18,802,886 | 1,660,357 | 1,233,200 | 44.6 | 28.0 |
| **1995** | 81,539,000 | 16,107,724 | 19,158,899 | 1,686,809 | 1,258,004 | 45.3 | 28.4 |
| **1996** | 81,817,000 | 16,300,230 | 19,499,930 | 1,711,276 | 1,281,218 | 45.9 | 28.7 |
| **1997** | 82,012,000 | 16,516,525 | 19,814,323 | 1,738,984 | 1,304,221 | 46.6 | 29.2 |
| **1998** | 82,057,000 | 16,741,748 | 20,054,858 | 1,768,179 | 1,321,629 | 47.3 | 29.5 |
| **1999** | 82,037,000 | 16,986,070 | 20,254,220 | 1,799,911 | 1,336,752 | 48.1 | 30.0 |
| **2000** | 82,163,000 | 17,219,958 | 20,409,568 | 1,830,796 | 1,348,941 | 48.7 | 30.3 |
| **2001** | 82,260,000 | 17,408,619 | 20,512,534 | 1,856,204 | 1,357,772 | 49.2 | 30.5 |
| **2002** | 82,440,000 | 17,585,111 | 20,572,800 | 1,879,730 | 1,363,939 | 49.5 | 30.7 |
| **2003** | 82,537,000 | 17,754,042 | 20,615,923 | 1,903,340 | 1,368,628 | 49.8 | 30.8 |
| **2004** | 82,532,000 | 17,933,798 | 20,652,745 | 1,928,085 | 1,373,193 | 50.3 | 31.1 |
| **2005** | 82,501,000 | 18,087,964 | 20,684,473 | 1,949,557 | 1,377,292 | 50.7 | 31.3 |
| **2006** | 82,438,000 | 18,240,238 | 20,731,024 | 1,970,914 | 1,382,203 | 51.1 | 31.5 |
| **2007** | 82,315,000 | 18,365,864 | 20,766,359 | 1,988,835 | 1,386,335 | 51.4 | 31.7 |
| **2008** | 82,218,000 | 18,461,934 | 20,805,953 | 2,002,844 | 1,390,556 | 51.8 | 31.9 |
| **2009** | 82,002,000 | 18,545,943 | 20,844,525 | 2,015,181 | 1,394,638 | 52.2 | 32.1 |
| **2010** | 81,802,000 | 18,162,046 | 20,542,952 | 2,121,200 | 1,418,431 | 55.3 | 32.7 |
| **2011** | 81,752,000 | 18,252,882 | 20,596,184 | 2,134,694 | 1,423,436 | 55.6 | 32.8 |
| **2012** | 80,328,000 | 18,351,090 | 20,665,140 | 2,149,361 | 1,429,779 | 56.9 | 33.6 |
| **2013** | 80,524,000 | 18,452,231 | 20,743,263 | 2,164,467 | 1,436,835 | 57.1 | 33.7 |
| **2014** | 80,767,000 | 18,557,401 | 20,850,325 | 2,180,232 | 1,446,271 | 57.3 | 33.8 |
| **2015** | 81,198,000 | 18,658,494 | 20,961,863 | 2,195,537 | 1,456,026 | 57.4 | 33.9 |
| **2016** | 82,176,000 | 18,762,486 | 21,086,208 | 2,211,256 | 1,466,627 | 57.2 | 33.7 |
| **2017** | 82,522,000 | 18,866,038 | 21,222,311 | 2,226,956 | 1,478,065 | 57.3 | 33.8 |
| **2018** | 82,792,000 | 18,966,444 | 21,369,968 | 2,242,305 | 1,490,084 | 57.6 | 34.0 |

## Adjusted building volume

Using building volume as a covariate for gridded population does not account for the fact that population density can be higher in multi-family dwellings than in single-family dwellings. Census data confirms that living floor area per capita is different in buildings of those categories (refer to main text), and different assumptions on this are accounted for in the bottom-up population mapping approach. In our top-down approach, we employed a sensitivity analysis and tested a variety of weighting factors to increase building volume of multi-family buildings and, thus, the population that is redistributed to that building type. Even though different weighing factors for multi-family buildings perform best at some scales and regarding some quality metrics, it turned out that a volume weighting factor of 1.6 yields best results with regard to most quality metrics at NUTS-3- and LAU-level. WD-VOLADJ1.6 is, thus, the model which was used for the comparison to other models in the main study.

**Table SI 6** Prediction **q**uality of models using different volume weighting factors for multi-family buildings.

| *Slope*  *R²*  *RMSE*  *MAE*  *MAPE* | **WD-VOLADJ 1.2** | **WD-VOLADJ 1.4** | **WD-VOLADJ 1.6** | **WD-VOLADJ 1.8** | **WD-VOLADJ 2.0** |
| --- | --- | --- | --- | --- | --- |
| **NUTS-1** | 1.05  0.98  736,973  559,229  14.55 | 1.05  0.98  756,830  553,387  13.43 | 1.04  0.98  792,105  571,689  13.32 | 1.04  0.98  829,127  586,728  13.19 | 1.04  0.97  870,508  601,994  13.13 |
| **NUTS-3** | 0.93  0.96  49,967  33,300  19.05 | 0.98  0.97  46,204  32,208  18.37 | 1.02  0.97  46,624  32,527  18.04 | 1.06  0.97  49,788  33,159  17.93 | 1.09  0.97  54,651  34,074  18.05 |
| **LAU** | 0.93  0.99  6,309  1,408  35.91 | 0.98  0.99  5,556  1,381  34.45 | 1.03  0.99  5,855  1,411  33.65 | 1.07  0.99  6,795  1,454  33.27 | 1.10  0.99  8,025  1,503  33.22 |
| **BPA** | 0.72  0.65  3,574  2,646  65.66 | 0.79  0.64  3,582  2,617  68.43 | 0.84  0.63  3,701  2,678  71.7 | 0.89  0.62  3,887  2,813  75.21 | 0.93  0.62  4,080  2,968  78.03 |

## Validation units and census population within REE ranges

**Table SI 7.1** Number of LAU units within ranges of REE using different gridded population models

| **REE value range** | **BD-BUILD** | **BD-RESI** | **WD-DENS** | **WD-VOL** | **WD-VOLADJ** | **BU-LFA** |
| --- | --- | --- | --- | --- | --- | --- |
| **[-100,-50]** | 154 | 62 | 124 | 369 | 621 | 1004 |
| **]-50,-25]** | 989 | 543 | 728 | 1382 | 2111 | 2617 |
| **]-25,-10]** | 1249 | 846 | 998 | 1603 | 1882 | 2006 |
| **]-10,0]** | 1031 | 830 | 930 | 1184 | 1294 | 1333 |
| **]0,10]** | 1112 | 985 | 1014 | 1230 | 1177 | 990 |
| **]10,25]** | 1509 | 1562 | 1624 | 1661 | 1378 | 1180 |
| **]25,50]** | 1832 | 2409 | 2239 | 1794 | 1364 | 980 |
| **]50,100]** | 1876 | 2578 | 2315 | 1293 | 834 | 639 |
| **]100** | 1288 | 1237 | 1080 | 537 | 392 | 304 |

**Table SI 7.2** Census population within ranges of REE using different gridded population models

| **REE value range** | **BD-BUILD** | **BD-RESI** | **WD-DENS** | **WD-VOL** | **WD-VOLADJ** | **BU-LFA** |
| --- | --- | --- | --- | --- | --- | --- |
| **[-100,-50]** | 6,374,781 | 2,100,092 | 736,611 | 601,406 | 1,068,888 | 1,756,495 |
| **]-50,-25]** | 19,578,069 | 23,408,807 | 23,353,404 | 11,207,126 | 10,261,850 | 11,495,934 |
| **]-25,-10]** | 13,967,519 | 12,176,832 | 13,171,745 | 20,839,755 | 16,737,379 | 14,015,843 |
| **]-10,0]** | 8,306,254 | 7,909,988 | 8,793,907 | 15,077,135 | 13,210,232 | 15,569,279 |
| **]0,10]** | 7,552,312 | 8,402,756 | 7,981,757 | 10,894,456 | 17,408,468 | 12,965,058 |
| **]10,25]** | 9,333,850 | 9,769,651 | 10,853,181 | 12,015,896 | 14,358,032 | 17,039,464 |
| **]25,50]** | 9,088,909 | 10,725,295 | 10,292,866 | 8,540,016 | 7,171,353 | 7,880,920 |
| **]50,100]** | 6,082,293 | 6,877,418 | 6,449,702 | 3,276,165 | 2,318,404 | 1,850,071 |
| **]100** | 2,505,389 | 1,421,395 | 1,159,061 | 340,348 | 257,697 | 219,239 |

## Gridded population results


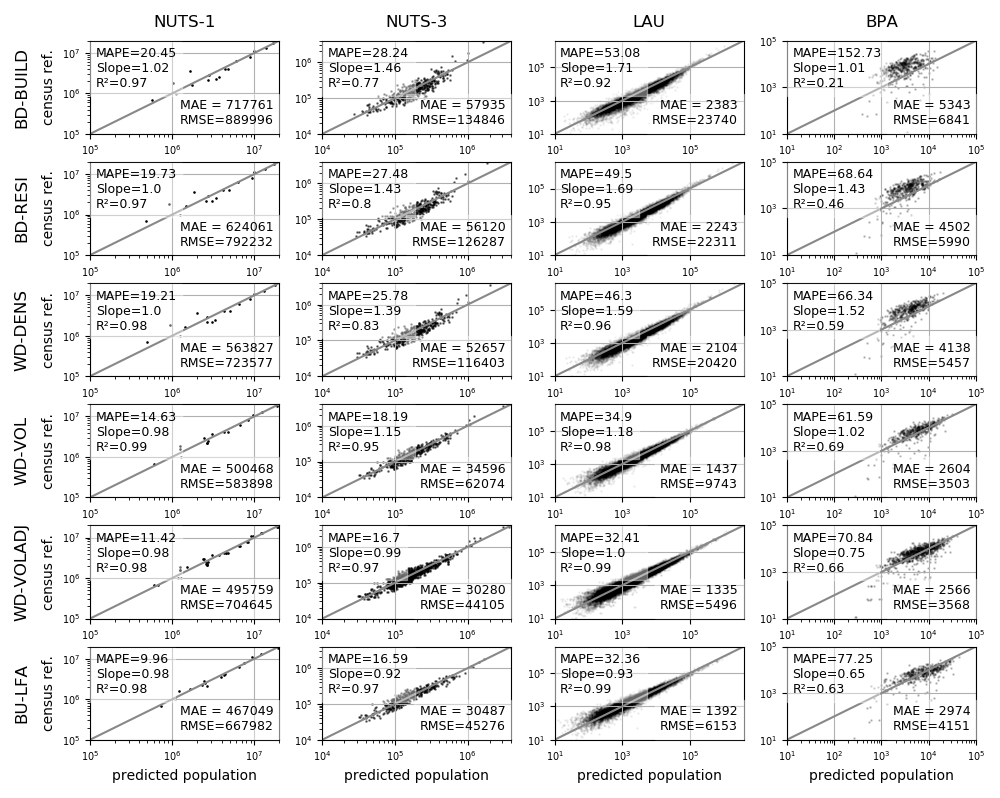


**Figure SI 8:** Population redistribution accuracies at different spatial validation scales (NUTS-1 to LAU and BPA) using the different approaches (BD-BUILD to WD-VOLADJ). Axes have logarithmic scale. Top-down binary models using building presence (BD-BUILD), type (BD-RESI). Weighted models using building density (BD-DENS), volume (BD-VOL) and adjusted volume (BD-VOLADJ). Bottom-up model using living floor area per capita (BU-LFA).

References

**1**. Schug F, Frantz D, Okujeni A, van der Linden S, Hostert P. Mapping urban-rural gradients of settlements and vegetation at national scale using Sentinel-2 spectral-temporal metrics and regression-based unmixing with synthetic training data. Remote Sensing of Environment. 2020; 246:111810. doi: 10.1016/j.rse.2020.111810.

**2**. Okujeni A, van der Linden S, Suess S, Hostert P. Ensemble Learning From Synthetically Mixed Training Data for Quantifying Urban Land Cover With Support Vector Regression. IEEE J Sel Top Appl Earth Observations Remote Sensing. 2017; 10:1640–50. doi: 10.1109/JSTARS.2016.2634859.

**3**. Schug F, Frantz D, Okujeni A, van der Linden S, Hostert P. Land cover fraction map of Germany at 10m spatial resolution based on Sentinel-1 and Sentinel-2 spectral temporal metrics. PANGAEA - Data Publisher for Earth & Environmental Science; 2020.

**4**. Müller H, Rufin P, Griffiths P, Barros Siqueira AJ, Hostert P. Mining dense Landsat time series for separating cropland and pasture in a heterogeneous Brazilian savanna landscape. Remote Sensing of Environment. 2015; 156:490–9. doi: 10.1016/j.rse.2014.10.014.

**5**. Potapov PV, Turubanova SA, Tyukavina A, Krylov AM, McCarty JL, Radeloff VC, et al. Eastern Europe's forest cover dynamics from 1985 to 2012 quantified from the full Landsat archive. Remote Sensing of Environment. 2015; 159:28–43. doi: 10.1016/j.rse.2014.11.027.

**6**. Geofabrik Download Server. Geofabrik 2020 [cited 30 Jan 2020]. Available from: https://download.geofabrik.de.

**7**. Federal Stat. Office, editor. Gesamtlänge der Straßen des überörtlichen Verkehrs in Deutschland von 1995 bis 2019 (in 1.000 Kilometer ) [Graph]. 2019 [cited 14 Sep 2020]. Available from: https://de.statista.com/statistik/daten/studie/2990/umfrage/gesamtlaenge-der-strassen-des-ueberoertlichen-verkehrs/.

**8**. Frantz D, Schug F, Okujeni A, Navacchi C, Wagner W, van der Linden S, et al. National-scale mapping of building height using Sentinel-1 and Sentinel-2 time series. Remote Sensing of Environment. 2021; 252:112128. doi: 10.1016/j.rse.2020.112128.

**9**. Frantz D. FORCE—Landsat + Sentinel-2 Analysis Ready Data and Beyond. Remote Sensing. 2019; 11:1124. doi: 10.3390/rs11091124.

**10**. Verdonck M-L, Okujeni A, van der Linden S, Demuzere M, Wulf R de, van Coillie F. Influence of neighbourhood information on ‘Local Climate Zone’ mapping in heterogeneous cities. International Journal of Applied Earth Observation and Geoinformation. 2017; 62:102–13. doi: 10.1016/j.jag.2017.05.017.

**11**. Bechtel B, Alexander P, Böhner J, Ching J, Conrad O, Feddema J, et al. Mapping Local Climate Zones for a Worldwide Database of the Form and Function of Cities. IJGI. 2015; 4:199–219. doi: 10.3390/ijgi4010199.

**12**. Rosentreter J, Hagensieker R, Waske B. Towards large-scale mapping of local climate zones using multitemporal Sentinel 2 data and convolutional neural networks. Remote Sensing of Environment. 2020; 237:111472. doi: 10.1016/j.rse.2019.111472.

**13**. Tucker CJ. Red and photographic infrared linear combinations for monitoring vegetation. Remote Sensing of Environment. 1979; 8:127–50. doi: 10.1016/0034-4257(79)90013-0.

**14**. Zha Y, Gao J, Ni S. Use of normalized difference built-up index in automatically mapping urban areas from TM imagery. International Journal of Remote Sensing. 2003; 24:583–94. doi: 10.1080/01431160304987.

**15**. Xu H. Modification of normalised difference water index (NDWI) to enhance open water features in remotely sensed imagery. International Journal of Remote Sensing. 2006; 27:3025–33. doi: 10.1080/01431160600589179.

**16**. Breiman L. Random Forests. Machine Learning. 2001; 45:5–32. doi: 10.1023/A:1010933404324.

**17**. Olofsson P, Foody GM, Herold M, Stehman SV, Woodcock CE, Wulder MA. Good practices for estimating area and assessing accuracy of land change. Remote Sensing of Environment. 2014; 148:42–57. doi: 10.1016/j.rse.2014.02.015.

**18**. Census db. from Fed. Stat. Offices, editor. Zensus 2011. Bayerisches Landesamt für Statistik 2011 [cited 23 Jun 2020]. Available from: https://ergebnisse.zensus2011.de/?locale=en#Home:

**19**. Gebäude und Wohnungen. Bestand an Wohnungen und Wohngebäuden, Bauabgang von Wohnungen und Wohngebäuden, Lange Reihen ab 1969 - 2018. ; 2019.

**20**. Benediktsson JA, Pesaresi M, Arnason K. Classification and feature extraction for remote sensing images from urban areas based on morphological transformations. IEEE Trans Geosci Remote Sensing. 2003; 41:1940–9. doi: 10.1109/tgrs.2003.814625.

**21**. Census db. from Fed. Stat. Offices. Gebäude- und Wohnungsbestand in Deutschland. ; 2014.
